# Comparative real-world safety profiles of caspofungin, micafungin, and anidulafungin: a disproportionality analysis based on FAERS and VigiAccess databases

**DOI:** 10.3389/fphar.2026.1840717

**Published:** 2026-06-18

**Authors:** Jingxiu Chen, Chunhua Chen, Xiaobin Lin, Jiajia Yan, Yifan Zheng, Pan Chen, Jia Li

**Affiliations:** 1 Department of Pharmacy, The First Affiliated Hospital of Sun Yat-sen University, Guangzhou, China; 2 Department of Clinical Pharmacy Translational Science, University of Michigan College of Pharmacy, Ann Arbor, MI, United States; 3 Department of Pharmacy, Guangxi Hospital Division of the First Affiliated Hospital, Sun Yat-sen University, Nanning, China

**Keywords:** adverse event, anidulafungin, caspofungin, disproportionality analysis, micafungin

## Abstract

**Background:**

Caspofungin, micafungin, and anidulafungin are three commonly used echinocandins recommended for the treatment of invasive candidiasis. This study aimed to comprehensively evaluate the safety profiles of these three agents to assist clinicians in making appropriate therapeutic decisions.

**Methods:**

A cross-sectional pharmacovigilance study based on the FDA Adverse Event Reporting System (FAERS) database was performed. Adverse event (AE) reports were collected from January 2004 to March 2025. The reporting odds ratio, proportional reporting ratio, and Bayesian confidence propagation neural network were used to identify, assess, and compare AE signals of the three echinocandins. Data from the VigiAccess database were used for external validation.

**Results:**

A total of 2343, 2338, and 406 cases associated with caspofungin, micafungin, and anidulafungin, respectively, were extracted from the FAERS database. Caspofungin exhibited a tendency toward higher reporting frequencies of hepatobiliary disorders and skin and subcutaneous tissue disorders compared with micafungin and anidulafungin. Hepatobiliary signals observed for anidulafungin should be interpreted cautiously, as they may reflect confounding by indication related to preferential use in patients with underlying hepatic dysfunction rather than direct drug toxicity. A range of AEs that were not highlighted in the labels were identified. Logistic regression analyses indicated that skin and subcutaneous tissue disorders associated with caspofungin or micafungin were significantly associated with hospitalization reporting. Supplementary PT-level analyses further identified several clinically relevant AEs associated with hospitalization, including drug reaction with eosinophilia and systemic symptoms for caspofungin, renal failure for micafungin, and hepatic failure for anidulafungin. Higher disproportionality signals related to caspofungin and micafungin were observed in patients aged≥65 years and females. Additionally, the sensitivity analyses demonstrated that this study possessed good robustness. The median time-to-onset of AEs was significantly longer for caspofungin at 4 days (IQR: 0–11 days) than for micafungin at 2 days (IQR: 0–8 days, *P* < 0.001) and anidulafungin at 1 day (IQR: 0–10 days, *P* = 0.001). The results in VigiAccess were broadly consistent with FAERS findings.

**Conclusion:**

This study provides a comparative pharmacovigilance overview of the safety profiles of three echinocandins using FAERS and VigiAccess data. Further prospective studies are needed to validate these observations.

## 1 Introduction

Invasive fungal infections (IFIs) remain a significant global health issue with high morbidity and mortality rates (Chang et al., 2017; Ikuta et al., 2024). In recent years, the occurrence of IFIs has increased as a result of an increase in the number of immunosuppressed patients with AIDS, cancer, undergoing solid organ transplantation, or receiving corticosteroid therapies (Brown et al., 2012). Clinically available antifungal drugs for IFIs are primarily limited to three classes: polyenes, triazoles, and echinocandins (Chang et al., 2017). Echinocandin antifungal agents exert broad-spectrum antifungal activity by specifically inhibiting the synthesis of β-(1,3)-D-glucan in the fungal cell wall, thereby resulting in osmotic instability and cell death (Odds et al., 2003; Johnson, 2021; Kofla and Ruhnke, 2011). Owing to their excellent and unique drug target that is only present in fungi without affecting mammalian cells, echinocandins exhibit low toxicity in humans (Chang et al., 2017). Currently, echinocandin drugs approved by the U.S. Food and Drug Administration (FDA) include caspofungin, micafungin, and anidulafungin (Emri et al., 2013), which are recommended as the primary drugs of choice to treat invasive candidiasis according to the 2016 Infectious Diseases Society of America candidiasis guideline (Pappas et al., 2016). Caspofungin was the first echinocandin approved by the FDA in 2001 (US Food and Drug Administration, 2021a). Micafungin was first approved in Japan in 2002 and by the U.S. FDA in March 2005, followed by anidulafungin in 2006 (US Food and Drug Administration, 2021b; Wasmann et al., 2018; US Food and Drug Administration, 2020).

In premarketing studies, the primary adverse events (AEs) associated with echinocandins included elevated levels of alanine aminotransferase and aspartate aminotransferase, fever, local phlebitis, gastrointestinal disturbances, headache, and rash (Cheng et al., 2024; Denning, 2003). However, clinical trials are typically limited by small sample sizes, short observation periods, and strict eligibility criteria. One recently published study focused only on AEs associated with caspofungin (Li et al., 2025). Consequently, existing research is inadequate for assessing the post-marketing safety profiles of micafungin and anidulafungin, as well as for comparing the differences among the three echinocandins (caspofungin, micafungin, and anidulafungin). With the increasing use of echinocandins for antifungal therapy, the potential impact of AEs deserves attention. It is necessary to conduct post-marketing evaluations to detect new AEs and compare the risk of AEs among echinocandins in pharmacotherapy in real-world settings. The FDA Adverse Event Reporting System (FAERS) is a publicly available pharmacovigilance database for the post-marketing surveillance of approved drugs in the United States, which contains a large number of real AE reports from various sources and reflects the real-world occurrence of AEs (Zou et al., 2024; Pan et al., 2024). VigiAccess (https://vigiaccess.org/) is a web application that allows public access to VigiBase, the largest global pharmacovigilance database of individual case safety reports registered by the World Health Organization (WHO) (Van De Ven et al., 2020). It contains pharmacovigilance data from over 130 countries and comprises spontaneous reports submitted by national pharmacovigilance centers (Nishida et al., 2025). The WHO-VigiAccess and FAERS databases serve as valuable real-world resources that encompass a wide range of drug users. The large sample sizes and global scope of both databases support robust statistical comparisons, thus enhancing the reliability of findings. This study aimed to explore the potential safety issues of these major echinocandin antifungal agents and compare their differences in safety by analyzing post-marketing AE reports from the FAERS and VigiAccess databases, thereby assisting clinicians in identifying potential AEs more effectively and optimizing treatment choices. This cross-sectional study has been reported in line with the STROCSS guidelines (Agha et al., 2025).

## 2 Materials and methods

### 2.1 Data sources and collection

This cross-sectional pharmacovigilance study based on the FAERS database was conducted in accordance with the READUS-PV (Reporting of a Disproportionality Analysis for Drug Safety Signal Detection Using Individual Case Safety Reports in PharmacoVigilance) guideline for reporting disproportionality analysis (Fusaroli et al., 2024). All AEs were coded using the Medical Dictionary for Regulatory Activities (MedDRA) terminology for systematic categorization. According to MedDRA (version 28.0), each AE record is assigned a specific preferred term (PT), which is categorized into a system organ class (SOC) and linked to only one primary SOC. The primary SOC is utilized to categorize PTs (He et al., 2024; Qiao et al., 2024).

In this study, American Standard Code for Information Interchange (ASCII) report files were downloaded from the FAERS database for data mining and statistical analysis, focusing on AE signals. The data cover the period from January 2004 to March 2025. This study focused on AE reports related to three echinocandins: caspofungin, micafungin, and anidulafungin. Only AE reports that identified these drugs as “primary suspect” were included. Rezafungin, one kind of echinocandins, was not included in this study due to marketing approval in 2023, with fewer reported AEs (US Food and Drug Administration, 2023). To enhance the reliability of findings, duplicate reports in the FAERS database were removed according to FDA recommendations. CASEID, FDA_DT, and PRIMARYID were extracted from the DEMO table. For records with identical CASEIDs, the report with the most recent FDA_DT was retained; if both CASEID and FDA_DT were identical, the report with the highest PRIMARYID was retained (Qiao et al., 2024). This deduplication procedure was applied only to the DEMO table to identify the final version of each case. After deduplication, all PT-level AE records associated with the retained PRIMARYID were preserved for analysis.

To further ensure the accuracy of findings, an external validation was conducted using the VigiAccess database for echinocandin-related AEs. The data encompassed its entire history up to Q4 2024 from the VigiAccess database.

### 2.2 Disproportionality analysis

**FIGURE 1:**
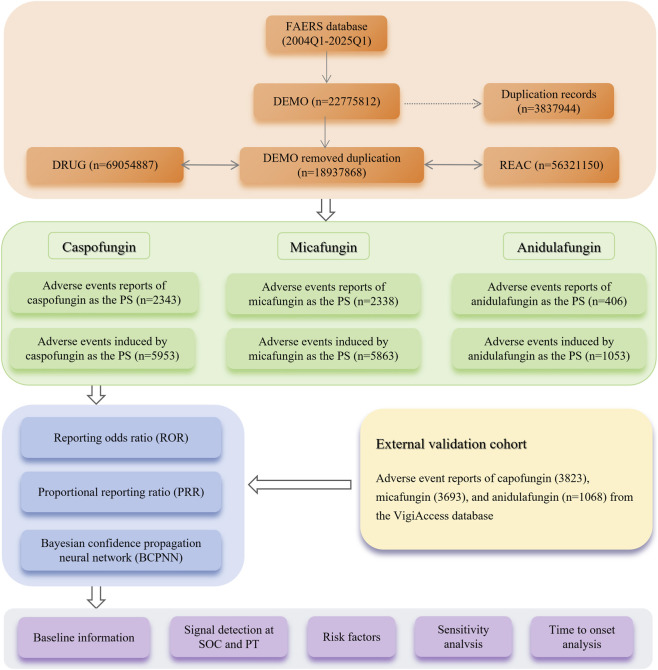
The flow chart of the study design. PS, primary suspect; SOC, system organ class; PT, preferred term.

A retrospective quantitative analysis was performed to obtain an in-depth understanding of the characteristics, patterns, similarities, and differences in real-world occurrences of AEs for the three echinocandin drugs. Based on the disproportionality analysis, three widely used signal detection methods were employed to identify potential AE signals: the reporting odds ratio (ROR), proportional reporting ratio (PRR), and Bayesian confidence propagation neural network (BCPNN) of information components (IC) (Bate et al., 1998; van Puijenbroek et al., 2002; Evans etal, 2001). ROR and PRR, classified as frequency-based approaches, are associated with high sensitivity but low specificity, whereas BCPNN is good at combining and cross-validating data from multiple sources, but with low sensitivity in signal detection (Yang, et al., 2024; Hu et al., 2025). All disproportionality analyses were performed based on a standard 2 × 2 contingency table (Supplementary Table S1). The corresponding formulas and criteria for positive signal detection are provided in Supplementary Table S2. To ensure the stability and reliability of the study results, a signal was considered positive only when all three disproportionality algorithms simultaneously met their predefined criteria. Here, *a* represents the number of reports containing both the target drug and the target adverse event. The criteria were defined as follows: (1) for ROR, *a* ≥ 3 and the lower limit of the 95% confidence interval (CI) > 1 (Van Puijenbroek et al., 2003); (2) for PRR, *a* ≥ 3, PRR ≥2, and χ^2^ ≥ 4 (Evans etal, 2001); and (3) for BCPNN, IC025 > 0, where IC025 represents the lower limit of the 95% CI of the information component (IC) (Noren et al., 2006). Beyond the threshold, a higher value of PRR/ROR/IC indicates stronger signal strength with a more robust association between the target drug and AE. The primary analysis of this study is presented in Figure 1.

### 2.3 Statistical analysis

Factors associated with hospitalization in echinocandin-related AE reports were analyzed using logistic regression analysis. Univariable logistic regression was initially performed, followed by multivariable analyses where appropriate. Independent variables included age (<65 vs. ≥65 years), sex, and AE categories at the SOC level, with hospitalization used as the sole dependent variable. Hospitalization was strictly defined as the presence or absence of the hospitalization outcome in FAERS records rather than a composite of all serious outcomes. Reports containing hospitalization were classified as hospitalization-positive. Because a single report may contain multiple coexisting serious outcomes (e.g., hospitalization and death), reports with hospitalization plus other outcomes were retained in the hospitalization-positive group if hospitalization was recorded, whereas reports without hospitalization were classified as hospitalization-negative. Second, variables with *P* < 0.05 in the univariate analysis were eligible for the multivariable logistic regression model to identify independent factors while adjusting for potential confounders. Multicollinearity was assessed using the variance inflation factor (VIF). The final model provided adjusted odds ratios (aORs) with 95% confidence intervals (CI), considering *P* < 0.05 as statistically significant. To further evaluate the heterogeneity within SOC-level analyses, supplementary exploratory logistic regression analyses were performed at the PT level using the 20 most frequently reported PTs for each echinocandin. PTs with *P* < 0.05 in univariate analyses were subsequently entered into multivariable logistic regression models adjusted for age and sex. All data processing, statistical analyses, and visualizations were performed using Microsoft Office Excel, SAS 9.4, SPSS 26.0, GraphPad Prism 10.1.2, and EVenn (Yang et al., 2024).

### 2.4 Sensitivity analysis

Subgroup analyses regarding age and sex in relation to echinocandin-associated AE signals were performed. The ROR algorithm was then applied. Based on a 2 × 2 contingency table, the chi-square test was employed for large samples with all expected frequencies of at least one, and Fisher’s exact test was used when the expected frequencies were less than one. Since reports from non-healthcare professionals could potentially confound the association between echinocandins and their adverse reactions, sensitivity analyses for the three echinocandins were carried out to verify the robustness of the results by restricting the reporting population to healthcare professionals.

### 2.5 Time-to-onset analysis

Time to onset was defined as the interval between treatment initiation and adverse event onset. Only reports containing complete and valid treatment initiation dates and event onset dates were included in the analysis. Reports with missing, incomplete, implausible, or chronologically inconsistent date records (e.g., event onset date earlier than treatment initiation date) were excluded from time-to-onset analyses. These data are presented as case numbers (percentages) and median values along with IQR. Differences in time-to-onset among the echinocandins were compared using the Breslow test (Generalized Wilcoxon), based on Kaplan-Meier analysis.

## 3 Results

### 3.1 Descriptive characteristics

After eliminating duplicates in the FAERS database, caspofungin was associated with 2,343 cases and 5,953 AEs, micafungin with 2,338 cases and 5,863 AEs, and anidulafungin with 406 cases and 1,053 AEs. The specific characteristics of the patients are shown in Table 1. In terms of sex distribution, males accounted for a higher proportion than females for all three medications, at 52.16% for caspofungin, 54.06% for micafungin, and 48.28% for anidulafungin. Patients aged >45 years accounted for the majority of AE reports. Notably, the majority of the reports were from health professionals, including physicians, pharmacists, and other health professionals, rather than consumers, which increased the credibility of this study. The total number of individual serious outcome events was greater than the number of serious cases, as a single patient report could record multiple coexisting serious outcomes simultaneously. Anidulafungin had the lowest number of reported AEs, likely due to its later market introduction and relatively lower clinical utilization (Supplementary Figure S1). The limited number of reports for anidulafungin may reduce the reliability of direct comparisons.

**TABLE 1:** Characteristics of adverse event reports related to three echinocandins from the FAERS database.

Characteristic	Caspofungin, N (%)	Micafungin, N (%)	Anidulafungin, N (%)
Case	2,343	2,338	406
Sex			
Female	832 (35.51)	830 (35.50)	132 (32.51)
Male	1222 (52.16)	1264 (54.06)	196 (48.28)
Not specifie	289 (12.33)	244 (10.44)	78 (19.21)
Age (years)			
<18	262 (11.18)	173 (7.40)	19 (4.68)
18–44	397 (16.94)	337 (14.41)	61 (15.02)
45–64	657 (28.04)	544 (23.27)	91 (22.41)
≥65	572 (24.41)	553 (23.65)	112 (27.59)
Not specified	455 (19.42)	731 (31.27)	123 (30.30)
Mean (SD)	50.04 (23.30)	52.60 (23.39)	55.06 (21.33)
Reporter			
Consumer	225 (9.60)	985 (42.13)	18 (4.43)
Lawyer	0	7 (0.30)	1 (0.25)
Not specified	66 (2.82)	39 (1.67)	33 (8.13)
Other health-professional	409 (17.46)	293 (12.53)	45 (11.08)
Pharmacist	536 (22.88)	282 (12.06)	99 (24.38)
Physician	1107 (47.25)	732 (31.31)	210 (51.72)
Serious report			
Serious	2,102 (89.71)	1740 (74.42)	388 (95.57)
Non-serious	241 (10.29)	598 (25.58)	18 (4.43)
Serious outcomes			
Life-threatening	254 (10.84)	159 (6.80)	52 (12.81)
Hospitalization-initial or prolonged	775 (33.08)	418 (17.88)	94 (23.15)
Disability	71 (3.03)	33 (1.41)	3 (0.74)
Death	936 (39.95)	754 (32.25)	140 (34.48)
Congenital anomaly	1 (0.04)	2 (0.09)	0 (0.00)
Required intervention	7 (0.30)	5 (0.21)	1 (0.25)
Other	1033 (44.09)	1042 (44.57)	236 (58.13)
Report region			
Europe	1023 (43.66)	432 (18.48)	193 (47.54)
Asia	586 (25.01)	945 (40.42)	64 (15.76)
North America	416 (17.76)	886 (37.90)	81 (19.95)
Not specified	200 (8.54)	30 (1.28)	1 (0.25)
Africa	78 (3.33)	25 (1.07)	21 (5.17)
South America	23 (0.98)	14 (0.60)	39 (9.61)
Oceania	17 (0.73)	6 (0.26)	7 (1.72)

Individual serious outcome counts are not mutually exclusive. One case may have multiple serious outcomes, so their sum exceeds the total number of serious reports.

### 3.2 Disproportionality analysis

#### 3.2.1 Analysis of AEs at the SOC level

Drug-unrelated AE signals were excluded from further analyses in the FAERS database (Supplementary Table S3). These signals were related to primary diseases (e.g., infection), drug use error events (e.g., product use issues, product storage errors, and off-label use), surgical and medical procedures (e.g., stem cell transplants), and other causes (e.g., drug resistance). After excluding drug-unrelated signals, 121, 123, and 50 positive signals were identified for caspofungin, micafungin, and anidulafungin, respectively.

**FIGURE 2:**
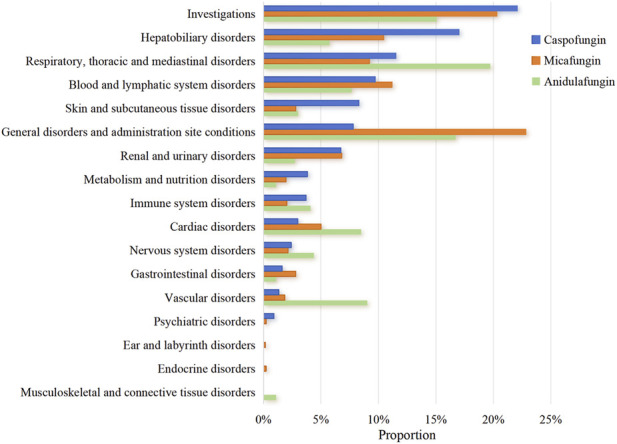
Distribution of adverse events associated with the three echinocandins by system organ classes from the FAERS database.

The proportion of positive signals for echinocandin-related AEs at the SOC level is shown in Figure 2. It was worth noting that the AE-involved SOCs of anidulafungin were apparently inconsistent with those of caspofungin and micafungin. Some of these findings align with SOCs associated with common adverse reactions in drug labels, thereby substantiating the reliability of these data. In addition, two novel SOC-level signals for micafungin were identified, which were not previously documented in clinical trials or drug inserts: endocrine disorders (4, 0.22%) with a single PT of inappropriate antidiuretic hormone secretion (ROR: 4.29) and ear and labyrinth disorders (3, 0.17%) with a single PT of ototoxicity (ROR: 17.21) (Supplementary Table S5).

#### 3.2.2 Analysis of AEs at the PT level

The top 30 AEs with the highest frequencies were identified (Table 2). AEs that were not highlighted in the drug labels for the three echinocandin drugs were identified. A detailed summary of the positive PTs is presented in Supplementary Tables S4–S6. The highest ROR signals (each unique to its respective drug) were: cutaneous calcification with caspofungin, intravascular haemolysis with micafungin, and haemodynamic instability with anidulafungin.

**TABLE 2:** Top 30 adverse events with the highest frequency for caspofungin, micafungin, anidulafungin at the PT level from the FAERS database.

No.	Caspofungin			Micafungin			Anidulafungin		
	PT	N	ROR (95%CI lower)	PT	N	ROR (95%CI lower)	PT	N	ROR (95%CI lower)
1	Multiple organ dysfunction syndrome*	111	26.24 (21.74)	Death*	204	2.57 (2.24)	Death*	39	2.74 (1.99)
2	Respiratory failure	60	8.52 (6.61)	Multiple organ dysfunction syndrome*	92	22.00 (17.90)	Dyspnoea	28	2.96 (2.03)
3	Blood alkaline phosphatase increased	47	19.12 (14.34)	Pyrexia	68	2.08 (1.63)	Multiple organ dysfunction syndrome	22	29.40 (19.27)
4	Renal failure	46	3.47 (2.60)	Aspartate aminotransferase increased	52	10.28 (7.82)	Respiratory failure*	13	10.46 (6.05)
5	Drug reaction with eosinophilia and systemic symptoms*	45	16.80 (12.53)	Respiratory failure	50	7.20 (5.45)	Thrombocytopenia	13	6.97 (4.04)
6	Alanine aminotransferase increased	45	7.58 (5.65)	Alanine aminotransferase increased	48	8.21 (6.18)	Tachycardia*	12	8.00 (4.53)
7	Cholestasis*	44	24.35 (18.10)	Hepatic function abnormal	43	12.63 (9.35)	Hypotension	11	3.24 (1.79)
8	Aspartate aminotransferase increased	41	7.96 (5.86)	Renal failure	41	3.14 (2.31)	Erythema	11	3.08 (1.70)
9	Acute kidney injury	38	2.01 (1.46)	Blood bilirubin increased	32	12.30 (8.69)	Seizure	10	3.40 (1.82)
10	C-reactive protein increased*	32	9.43 (6.66)	Liver disorder	31	7.49 (5.26)	Acute kidney injury*	10	3.00 (1.61)
11	Gamma-glutamyltransferase increased	31	14.12 (9.92)	Cardiac failure*	31	4.07 (2.86)	Hepatic failure	9	17.48 (9.07)
12	Hepatic function abnormal	31	8.94 (6.28)	Platelet count decreased	28	2.77 (1.91)	Anaphylactic reaction	8	8.90 (4.44)
13	Pancytopenia*	29	5.46 (3.79)	Disseminated intravascular coagulation	27	19.80 (13.56)	Oxygen saturation decreased*	8	8.72 (4.35)
14	Graft versus host disease	28	39.67 (27.35)	Hepatic failure	27	9.38 (6.43)	Bradycardia*	8	8.71 (4.34)
15	Blood lactate dehydrogenase increased*	28	17.55 (12.11)	Blood creatinine increased	27	4.30 (2.95)	Hypertension	8	2.23 (1.11)
16	Hepatic failure	28	9.59 (6.61)	Renal impairment	27	3.46 (2.37)	Bronchospasm	7	28.39 (13.50)
17	Eosinophilia*	27	16.45 (11.27)	Hypokalaemia	25	5.79 (3.91)	Cyanosis*	7	25.82 (12.28)
18	Blood bilirubin increased	27	10.21 (6.99)	Cardiac arrest	25	3.14 (2.12)	Anaphylactic shock	7	16.54 (7.87)
19	White blood cell count increased*	27	7.12 (4.88)	General physical health deterioration*	23	2.21 (1.47)	Hypoxia*	6	10.23 (4.58)
20	Hypokalaemia	27	6.16 (4.22)	White blood cell count decreased	22	2.13 (1.40)	Pancytopenia	6	6.39 (2.87)
21	Acute respiratory distress syndrome*	24	14.13 (9.46)	Blood alkaline phosphatase increased	21	8.62 (5.62)	Hepatic enzyme increased	6	5.45 (2.44)
22	Agranulocytosis	24	13.96 (9.35)	Thrombocytopenia	21	2.01 (1.31)	Cardiac arrest*	6	4.20 (1.88)
23	Thrombocytopenia	24	2.26 (1.51)	Gastrointestinal haemorrhage	20	2.42 (1.56)	Acute hepatic failure	5	22.57 (9.37)
24	Rash maculo-papular*	23	10.98 (7.29)	Acute respiratory distress syndrome	19	11.34 (7.23)	Blood alkaline phosphatase increased	5	11.44 (4.75)
25	Febrile neutropenia	23	3.69 (2.45)	Drug-induced liver injury	18	6.86 (4.32)	Blood bilirubin increased	5	10.68 (4.44)
26	Drug-induced liver injury	22	8.26 (5.44)	Hepatic enzyme increased	18	2.93 (1.84)	Leukopenia	5	5.94 (2.47)
27	Liver disorder	21	4.99 (3.25)	Bone marrow failure*	17	8.46 (5.25)	Alanine aminotransferase increased	5	4.74 (1.97)
28	Disseminated intravascular coagulation	20	14.42 (9.29)	Renal disorder	17	3.83 (2.38)	Blood creatinine increased	5	4.43 (1.84)
29	Hepatocellular injury	20	12.17 (7.85)	Leukopenia	17	3.62 (2.25)	Cardiac failure*	5	3.65 (1.52)
30	Transaminases increased	20	9.37 (6.04)	Pancytopenia	17	3.25 (2.02)	Haemodynamic instability	4	32.57 (12.20)

PT, preferred term; CI, confidence interval; Asterisks (*) indicate unexpected signals that are not indicated in the drug labels.

#### 3.2.3 Comparison of AE signals

**FIGURE 3:**
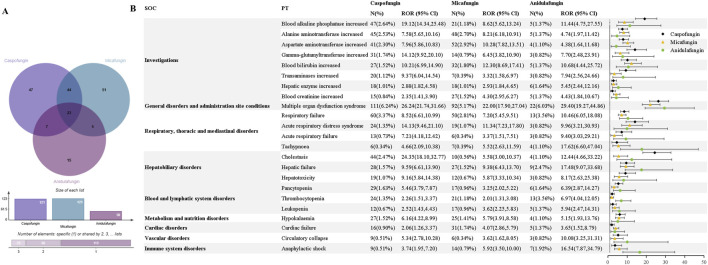
Comparative safety profiles of caspofungin, micafungin, and anidulafungin from the FAERS database. **(A)** Network Venn diagram of PT-positive signals for the three echinocandins. Numbers indicate the number of PT signals in each colored area. **(B)** The forest plot on the right displays the ROR values and their 95% confidence intervals for 23 overlapping PTs among the three echinocandins. PT, preferred term; SOC, system organ class; ROR, reporting odds ratio.

As shown in Figure 3, the comparative analysis of AE signals identified for the three echinocandin medications revealed 23 overlapping PTs across caspofungin, micafungin, and anidulafungin, such as multiple organ dysfunction syndrome, respiratory failure, blood alkaline phosphatase increased, alanine aminotransferase increased and cholestasis. Considering investigations related to hepatobiliary dysfunction and hepatobiliary disorders, caspofungin demonstrated the highest ROR for blood alkaline phosphatase increased, gamma-glutamyltransferase increased, transaminases increased, and cholestasis among these three echinocandins, whereas anidulafungin exhibited the highest ROR for hepatic failure.

**FIGURE 4:**
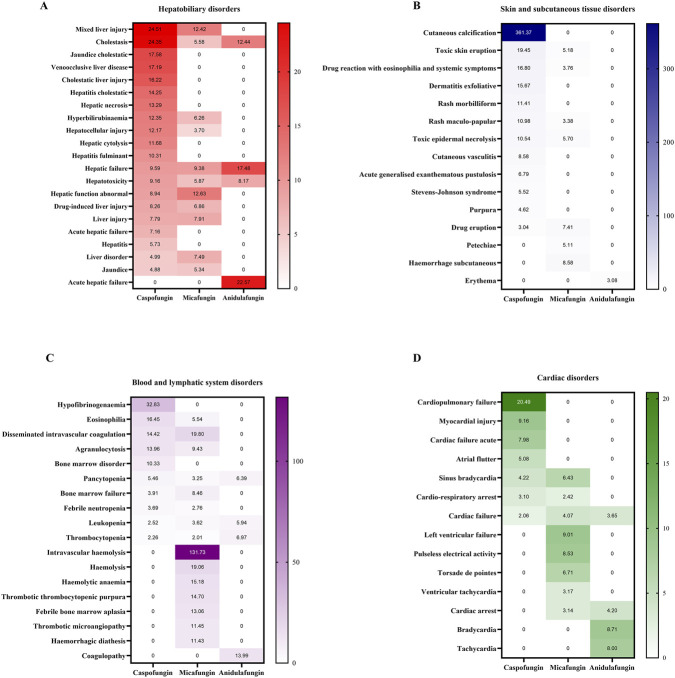
Comparison of positive signal strength of the adverse events for the three echinocandins ranked by ROR at four different system organ class levels from the FAERS database, including hepatobiliary disorders **(A)**, skin and subcutaneous tissue disorders **(B)**, blood and lymphatic system disorders **(C)** and cardiac disorders **(D)**. The x-axis of each figure represents caspofungin, micafungin, and anidulafungin, while the y-axis represents the preferred terms under the given system organ class.

In addition, heat maps were generated to compare the positive signal strength of the AEs for the three echinocandins ranked by ROR at four different SOC levels, including hepatobiliary disorders, skin and subcutaneous tissue disorders, blood and lymphatic system disorders, and cardiac disorders (Figure 4).

#### 3.2.4 Factors associated with hospitalization in echinocandin-related AE reports

**FIGURE 5:**
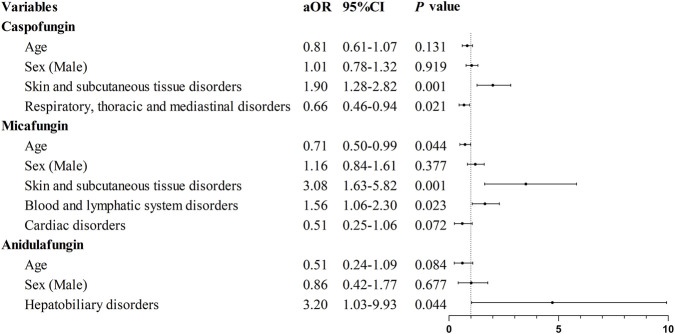
Factors associated with hospitalization outcome in the FAERS database. Forest plot of multivariate logistic regression analysis.

Logistic regression was performed to identify the factors (including age, sex, and SOCs) associated with hospitalization among patients receiving echinocandins (Figure 5). The skin and subcutaneous tissue disorders of caspofungin were identified to be associated with hospitalization reporting (aOR: 1.90; 95% CI, 1.28–2.82). Micafungin had two SOCs associated with hospitalization reporting, including skin and subcutaneous tissue disorders (aOR: 3.08; 95% CI, 1.63–5.82) and blood and lymphatic system disorders (aOR: 1.56; 95% CI, 1.06–2.30), while anidulafungin-related hepatobiliary disorders were identified to be associated with hospitalization reporting (aOR: 3.20; 95% CI, 1.03–9.93). Analysis of all SOCs was adjusted for the influence of age and sex through the logistic regression model. Collinearity diagnostics showed a corrected VIF <2 for all variables, indicating the absence of relevant multicollinearity. Supplementary exploratory analyses at the PT level were conducted using the 20 most frequently reported PTs for each echinocandin (Supplementary Table S7). Several clinically relevant PTs were found to be associated with hospitalization reporting, including drug reaction with eosinophilia and systemic symptoms for caspofungin, renal failure for micafungin, and hepatic failure for anidulafungin.

#### 3.2.5 Sensitivity analysis

**FIGURE 6:**
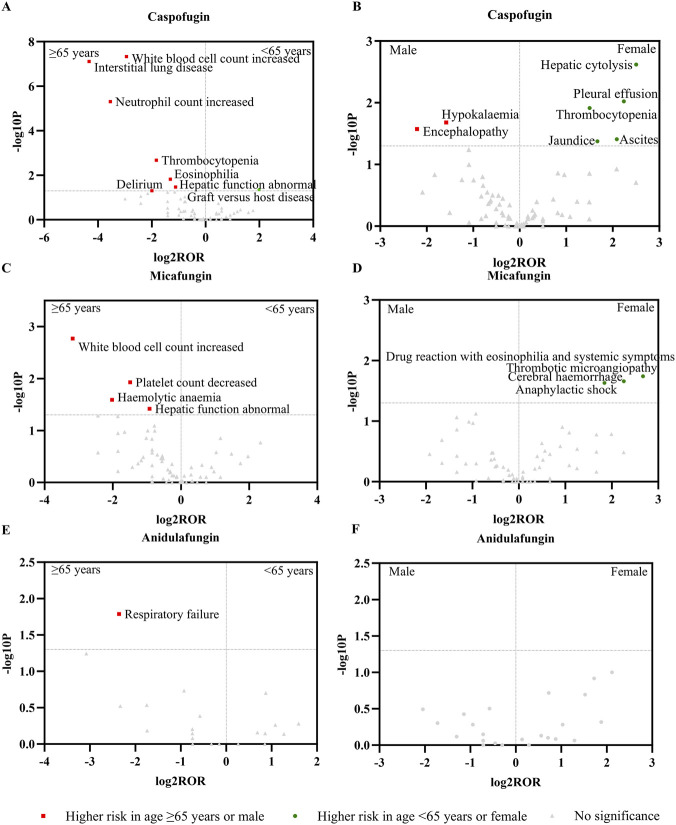
Volcano plot for difference detection of positive signals for echinocandins at the preferred term level from the FAERS database. Panels A/C/E are for age, and B/D/F are for sex. **(A)** Age differences between patients with age <65 years and ≥65 years for caspofungin. **(B)** Sex differences between females and males for caspofungin. **(C)** Age differences between patients with age <65 years and ≥65 years for micafungin. **(D)** Sex differences between females and males for micafungin. **(E)** Age differences between patients with age <65 years and ≥65 years for anidulafungin. **(F)** Sex differences between females and males for anidulafungin. The X-axis is the logarithm of the ROR value (log2ROR) based on the ROR algorithm, and the Y-axis is the negative logarithm of the P-value calculated using chi-square test and Fisher’s exact test (−log10 P). The individual points represent different preferred term signals. Red points indicate potential adverse events in patients with age ≥65 years or males, while green points denote those in patients with age <65 years or females. ROR, reporting odds ratio.

Volcano plots were constructed to illustrate the differences in positive signals with respect to age and sex for echinocandins. As shown in Figure 6, some signals exhibited distinct characteristics based on age and gender. Notably, a higher disproportionality signals for some AEs was observed in patients over 65 years of age and females who were treated with caspofungin or micafungin.

**FIGURE 7:**
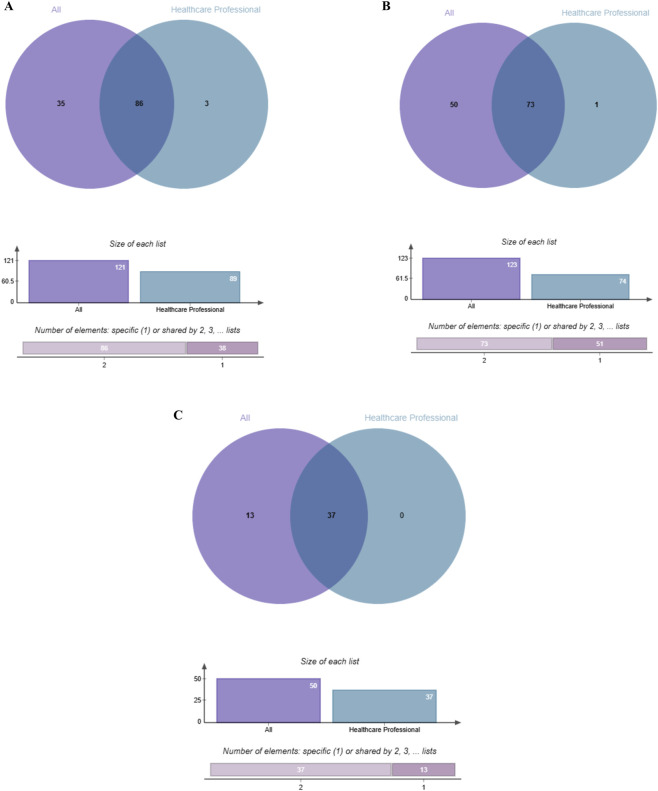
Network Venn diagram of sensitivity analyses performed by restricting the reporting population to healthcare professional from the FAERS database. Numbers indicate the number of preferred term signals in each colored area. **(A)** Caspofungin; **(B)** Micafungin; **(C)** Anidulafungin.

To eliminate reporting bias mediated by the reporting population, subsequent sensitivity analyses of the three echinocandins were conducted (Figure 7; Supplementary Table S8). Among the three echinocandins, pharmacovigilance data from healthcare professionals were similar to those from all reported populations. Thus, the sensitivity analysis revealed that this study had good robustness.

**FIGURE 8:**
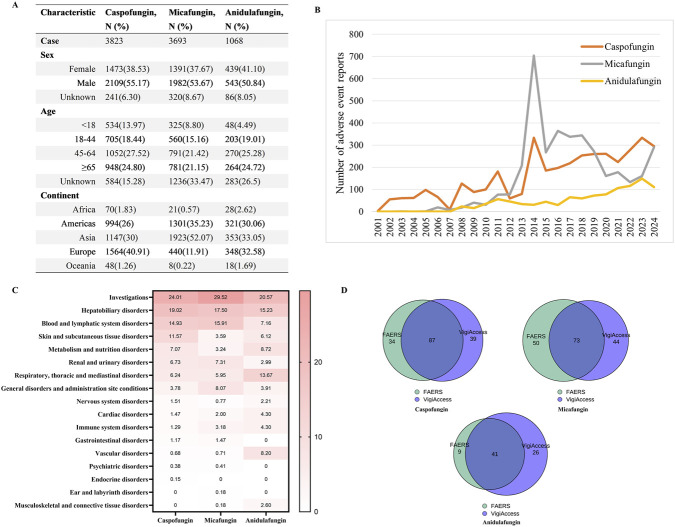
External validation from the VigiAccess database. **(A)** Characteristics of adverse event reports related to three echinocandins. **(B)** Number of annual reports of echinocandin-related adverse events. **(C)** Heat map of signal detection at the system organ class level. The numbers represented proportion. **(D)** Network Venn diagram of positive signals in the FAERS and VigiAccess database. Numbers indicate the number of preferred term signals in each colored area.

**FIGURE 9:**
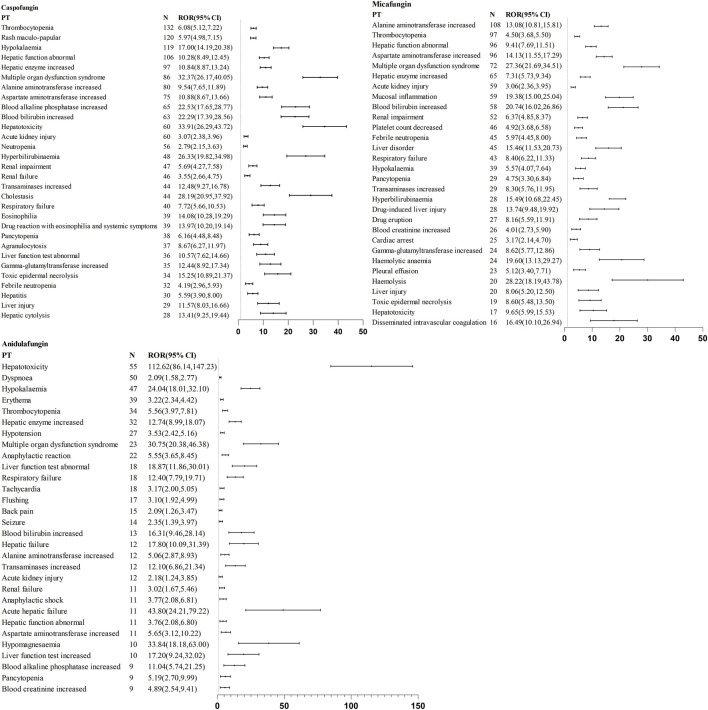
Signal detection at the PT level from the VigiAccess database. A forest plot representing ROR values along with their 95% confidence interval. PT, preferred term; ROR, reporting odds ratio.

To validate the reliability of FAERS database findings, the VigiAccess database was employed for comparison. The relevant results are presented in Figures 8, 9; Supplementary Tables S9–S11, which were broadly consistent with FAERS findings.

### 3.3 Time-to-onset analysis

As shown in Table 3, findings exhibited that a majority of patients experienced AEs within the first 30 days of treating echinocandins. The median times to onset of AEs associated with caspofungin, micafungin, and anidulafungin were 4 days (IQR: 0–11 days), 2 days (IQR: 0–8 days), and 1 day (IQR: 0–10 days), respectively. Significant differences were observed in the time-to-onset distributions among the three echinocandins (*P* < 0.001). Bonferroni correction was applied when comparing the echinocandins. These findings highlight the importance of monitoring for potential AEs during the initial phase of echinocandin therapy.

**TABLE 3:** Time to onset of adverse events associated with the three echinocandins from the FAERS database.

Time to onset (days)	Caspofungin, n = 893 (%)	Micafungin, n = 1026 (%)	Anidulafungin, n = 156 (%)
0–30	830 (92.95)	962 (93.76)	145 (92.95)
31–60	46 (5.15)	37 (3.61)	9 (5.77)
61–90	7 (0.78)	8 (0.78)	0 (0.00)
91–120	1 (0.11)	3 (0.29)	0 (0.00)
121–150	1 (0.11)	3 (0.29)	0 (0.00)
151–180	4 (0.45)	3 (0.29)	0 (0.00)
181–360	1 (0.11)	5 (0.49)	1 (0.64)
>360	3 (0.34)	5 (0.49)	1 (0.64)
Median (Q1, Q3)	4 (0,11)	2 (0, 8)^a^	1 (0, 10)^b^

^a^ caspofungin vs. micafungin, *P* < 0.001.

^b^ caspofungin vs. anidulafungin, *P* = 0.001.

## 4 Discussion

This study provides an in-depth analysis of the post-market safety profiles of caspofungin, micafungin, and anidulafungin in FAERS and VigiAccess databases. By comparing and analyzing the AEs of the three echinocandins, this study provided a thorough understanding of the similarities and differences in the safety profiles of these three agents, including common and unique AEs, thereby enabling more comprehensive safety evaluations and more precise clinical judgment in drug selection.

### 4.1 Hepatobiliary disorders

It is worth noting that caspofungin and micafungin are both metabolized by the liver, while anidulafungin is unique in that it is not hepatically metabolized (Denning, 2003; Chen et al, 2011). Anidulafungin undergoes slow chemical degradation in bile to form a ring-opened peptide that lacks antifungal activity (US Food and Drug Administration, 2020; Chen et al, 2011). Thus, the risk of liver injury caused by anidulafungin may be lower than that caused by caspofungin and micafungin. As indicated in the drug labels, dosage adjustment of caspofungin is needed for adult patients with moderate and severe hepatic impairment, whereas no dosing adjustments of micafungin and anidulafungin are required for patients with any degree of hepatic insufficiency (US Food and Drug Administration, 2020; US Food and Drug Administration, 2021a; US Food and Drug Administration, 2021b). In this study, hepatobiliary disorders and investigation abnormalities related to hepatobiliary function were observed among all three echinocandins in both databases. Caspofungin had the highest reporting frequency of hepatobiliary disorders, followed by micafungin and anidulafungin. The results were in line with those of previous studies and the drug labels mentioned above. Additionally, caspofungin exhibited notably stronger signals for both cholestasis and increased blood alkaline phosphatase levels than micafungin and anidulafungin. More hepatotoxicity in caspofungin was likely to be associated with the longer time that this agent had been available for use (Kauffman and Carver, 2008). Notably, a higher disproportionality signals of abnormal hepatic function was observed in patients over 65 years of age treated with caspofungin or micafungin in the FAERS database.

A large-scale retrospective cohort study initially suggested a higher risk of severe hepatotoxicity with anidulafungin, but after adjusting for confounders, no significant difference was found compared to caspofungin or micafungin. It further demonstrated that anidulafungin was predominantly used in a high-risk population with more severe baseline liver impairment and comorbidities (Vekeman et al., 2018). In this study, hepatobiliary disorder-related signals, including hepatic failure, were reported more frequently in patients receiving anidulafungin. However, these findings should be interpreted with substantial caution. Because anidulafungin is not hepatically metabolized (US Food and Drug Administration, 2020), it is commonly preferred in patients with pre-existing hepatic dysfunction or severe critical illness (Vekeman et al., 2018). Therefore, the observed hepatic failure signals are likely driven predominantly by the underlying liver disease of the treated population rather than direct hepatotoxicity attributable to anidulafungin itself. These findings should be considered descriptive pharmacovigilance signals rather than evidence of a causal relationship.

### 4.2 Skin and subcutaneous tissue disorders

In this study, caspofungin exhibited a higher reporting frequency of skin and subcutaneous tissue disorders than micafungin and anidulafungin in both databases. Logistic regression analysis revealed that skin and subcutaneous tissue disorders were associated with hospitalization reporting in patients treated with caspofungin and micafungin. Especially, caspofungin was unique in the signal of cutaneous calcification with the highest ROR, which was not present in patients exposed to micafungin and anidulafungin. Concurrently, cutaneous calcification for caspofungin is a novel signal that has not been documented in the drug insert. Cutaneous calcification is a disorder characterized by deposition of calcium salts in the skin and subcutaneous tissues (Reiter et al., 2011). A study reported four patients with toxic epidermal necrolysis exhibiting calcinosis cutis after treatment with caspofungin (Colboc et al., 2022), which is similar to the effect observed in this study. Caspofungin is an agonist of the ryanodine receptor, which is strongly expressed in keratinocytes (Denda et al., 2012; Koch et al., 2018). It might induce a dose-dependent increase in intracellular calcium concentration in keratinocytes via the activation of ryanodine receptors, thereby leading to delayed cutaneous healing (Colboc et al., 2022; Koch et al., 2018). Additionally, regarding toxic epidermal necrolysis, caspofungin showed a stronger signal than micafungin (ROR 10.54 vs. ROR 5.70 in FAERS; ROR 15.25 vs. ROR 8.60 in VigiAccess). However, the cutaneous calcinosis signal identified for caspofungin should be interpreted cautiously because it was based on only a few reports and may be secondary to severe skin necrosis rather than direct drug toxicity. Further clinical validation is warranted.

To our knowledge, this is the first study to review skin and subcutaneous tissue disorders following echinocandin treatment. Special attention should be paid to skin and subcutaneous tissue disorders induced by caspofungin, with an emphasis on early recognition and monitoring of cutaneous calcification.

### 4.3 Blood and lymphatic system disorders

It is reported that the number of AEs associated with haemolysis was similar between micafungin and caspofungin (Pappas et al., 2007). However, this study indicated that only micafungin exhibited positive signals for intravascular haemolysis, haemolysis, and haemolytic anaemia in both the databases. This result is consistent with the FDA-approved labeling information of the three echinocandins (US Food and Drug Administration, 2020; US Food and Drug Administration, 2021a; US Food and Drug Administration, 2021b). Hence, careful monitoring of hemolysis after micafungin administration is necessary. In addition, it was observed that common AE signals in blood and lymphatic system disorders associated with the three echinocandins encompassed pancytopenia, leukopenia, and thrombocytopenia, which were identified as stronger signals in anidulafungin than in caspofungin and micafungin. Clinicians should consider these signal strengths when selecting echinocandins for fungal infection therapy in patients at risk for blood and lymphatic system disorders. Moreover, blood and lymphatic system disorders were identified to be associated with hospitalization reporting in the course of treatment with micafungin (aOR: 1.56) in the FAERS database. Stronger signals for decreased platelet count and haemolytic anaemia were identified among patients aged ≥65 years. For caspofungin, stronger disproportionality signals for thrombocytopenia were observed among patients aged ≥65 years and females. A comprehensive strategy integrating age and sex factors is essential for the prevention, early detection, and management of blood and lymphatic system disorders to minimize patient harm.

### 4.4 Cardiac disorders

Cardiac AEs such as cardiac failure, cardiac arrest, cardio-respiratory arrest, and sinus bradycardia were also notable. 1n the FAERS database, anidulafungin was associated with the highest reporting frequency of cardiac disorders (8.49%, 31/1780), followed by micafungin (89, 5.00%) and caspofungin (53, 2.98%), aligning with the result of VigiAccess database. For anidulafungin, the most unexpected signals at the PT level were related to cardiac disorders. *In vivo* studies have claimed that administration of anidulafungin or caspofungin induces a dose-dependent decrease in cardiac contractility and a dose-dependent increase in cytotoxicity in cardiac myocytes, whereas micafungin has no significant impact on cardiac function (Koch et al., 2015; Koch et al., 2016; Arens et al., 2014). Evidences suggest that mitochondrial toxicity is the underlying mechanism of echinocandin-related cardiac dysfunction (Cleary and Stover, 2015; Stover et al., 2014). It is possible that the difference in cardiac toxicity was a result of the lipophilicity of these agents. The more lipophilic agents (anidulafungin and caspofungin) are more toxic than those with high hydrophilicity (micafungin) (Koch et al., 2015; Stover et al., 2014a; Stover et al., 2014b). Three clinical case reports revealed the possible impact of caspofungin and anidulafungin treatment on cardiac function (Lichtenstern et al., 2013). Nevertheless, cases from the FAERS database indicated that micafungin was associated with cardiac disorders characterized by left ventricular failure, pulseless electrical activity, and torsade de pointes. Among the three echinocandins, only anidulafungin exhibited significant hemodynamic instability, with the strongest ROR of 32.57 from the FAERS database. Previous cases have reported that intensive care unit patients developed hemodynamic instability following the administration of anidulafungin (Lichtenstern et al., 2013; Fink et al., 2013), which is in line with this study. All three echinocandins have cardiac AE reports. No clear superiority can be determined at present. Enhanced monitoring is recommended for any echinocandin in patients with pre-existing cardiac disease. Prospective and observational trials are needed to further investigate the population at the greatest risk of cardiotoxic reactions to echinocandins.

### 4.5 Novel safety signals requiring further investigation

Several unexpected AE signals were identified in the present study, which may provide additional hypotheses for future pharmacovigilance research. However, these findings should be interpreted cautiously because spontaneous reporting databases are inherently subject to underreporting, reporting bias, missing clinical information, and confounding by indication or concomitant medications. Among these signals, syndrome of inappropriate antidiuretic hormone secretion (SIADH) was detected as a potential signal for micafungin. Although this association has not been prominently reported in current prescribing information, the absolute number of reports was very limited. In addition, SIADH may be influenced by multiple clinical factors, including central nervous system disorders, pulmonary diseases, malignancy, and concomitant medications such as antidepressants or diuretics. Therefore, this finding should be regarded as hypothesis-generating only and requires further validation in prospective studies or well-designed real-world investigations before any causal interpretation or modification of current prescribing information can be considered. Other newly identified signals should likewise be interpreted conservatively until supported by additional clinical evidence or independent pharmacovigilance studies.

### 4.6 Subgroup analysis conducted by age and sex

Older individuals using antimicrobials have been reported to have a higher risk of AEs owing to age-related changes in pharmacokinetics and pharmacodynamics, multimorbidity, and polypharmacy (Soraci et al., 2023). There is no consistent evidence that age is a general risk factor for drug-induced liver injury (DILI); however, DILI phenotypes might be influenced by age, making cholestatic damage and chronic DILI more likely to occur. Antimicrobials and cardiovascular drugs were the most likely medications to cause DILI in the elderly (Lucena et al., 2020). A systematic review and meta-analysis revealed that the overall absolute risks of acute kidney injury among older adults prescribed aminoglycosides and glycopeptides were 15.1% (95% CI: 12.8–17.3) and 19.1% (95% CI: 15.4–22.7) (Chinzowu et al., 2021). Hirai et al. (2022) highlighted that patients aged >65 years had a higher risk of nephrotoxicity associated with teicoplanin than those aged ≤65 years. However, there are no available data regarding the risk of AEs among different ages following echinocandin exposure. This study found higher disproportionality signals of white blood cell count increased and hepatic function abnormal among patients over 65 years of age treated with caspofungin or micafungin. For caspofungin, higher disproportionality reporting signals for interstitial lung disease, neutrophil count increased, and thrombocytopenia were identified among patients aged ≥65 years. For micafungin, stronger disproportionality signals for decreased platelet count and haemolytic anaemia were observed among patients aged ≥65 years. In summary, stronger disproportionality signals for several AEs associated with caspofungin and micafungin were observed among patients aged ≥65 years.

It has been shown that females generally have a higher risk of developing adverse drug reactions than males (Martin et al., 1998; De Vries et al., 2019). However, global post-marketing surveillance data on spontaneous reports indicated that females reported more adverse drug reactions than males (Watson et al., 2019). Therefore, research on sex differences in AEs may be subject to reporting bias caused by sex. Moreover, one study demonstrated that there was no difference between females and males in the proportion of severe AEs associated with hospital admissions (Hendriksen et al., 2021). At present, there is no evidence to support significant sex differences in the AEs of echinocandins. In this study, males who received treatment with caspofungin exhibited a higher likelihood of experiencing hypokalaemia and encephalopathy, while females had a higher disproportionality signal of hepatic cytolysis, pleural effusion, and thrombocytopenia. For micafungin, females had a higher propensity for drug reaction with eosinophilia and systemic symptoms, thrombotic microangiopathy, cerebral haemorrhage, and anaphylactic shock than males. A higher disproportionality signal for several adverse events was observed among females treated with caspofungin and micafungin, while the majority of reported cases were in males in both the FAERS and VigiAccess databases. There was almost no difference in positive signals associated with anidulafungin in terms of age and sex. Because exposure denominators and sex-stratified prescribing volume were unavailable in the FAERS database, the observed sex-related differences may reflect reporting bias, differential drug utilization, or prescribing patterns rather than true biological differences. Therefore, these findings should be interpreted cautiously.

Time-to-onset findings should be interpreted cautiously because spontaneous reporting data are inherently subject to recall bias, reporting bias, and incomplete documentation. In addition, caspofungin was approved earlier than micafungin and anidulafungin, and earlier reports may be more susceptible to delayed reporting or recall-related bias. Differences in treatment indications, patient characteristics, hospital length of stay, and monitoring intensity across the three echinocandins may also have influenced the recorded onset intervals. Therefore, these findings should be interpreted descriptively as reporting characteristics rather than evidence of causal or intrinsic differences in adverse event onset among agents.

### 4.7 Strengths and limitations

This study has several strengths. Firstly, the FAERS and VigiAccess databases represent the most comprehensive repository of post-marketing safety profiles for pharmaceuticals. Secondly, this study identified the largest number of echinocandin-associated AEs to date. Unlike previous research (Li et al., 2025), a comprehensive understanding of the similarities and differences in the safety profiles of the three echinocandins was provided in this study. In addition, hospitalization-associated analyses were conducted using age, sex, and AE categories at the SOC levels for the three echinocandins. Supplementary PT-level analyses revealed substantial heterogeneity among individual PTs within the same SOC with respect to hospitalization-associated reporting. These findings indicate that SOC-level analyses provide a broad overview of organ-system involvement, whereas PT-level analyses may better identify clinically meaningful AEs associated with hospitalization. Moreover, differences in AE reports for the three echinocandins with respect to age and sex were further revealed. To our knowledge, this study is the first retrospective analysis based on both FAERS and VigiAccess databases to review and compare the comprehensive safety profiles of caspofungin, micafungin, and anidulafungin. Given the potential differences in the signals generated by healthcare professionals and consumers’ self-reporting, sensitivity analyses were conducted to indicate the stability and reliability of these findings.

Several limitations should be taken into account when interpreting the results of this study. Firstly, FAERS and VigiAccess are voluntary reporting systems that may lead to potential gaps in data quality, accuracy, or completeness. The two databases lack reliable denominator data, such as real-world prescribing volume or exposure duration stratified by sex. Therefore, the observed sex-specific differences in AE reporting could not be adjusted for drug utilization patterns or exposure person-time. Secondly, it is difficult to determine whether an AE is attributable to the target drug, underlying disease, or concomitant medications, thereby leading to an inaccurate representation of the true safety profile of the target drug. Death-related signals identified from spontaneous reporting systems should be interpreted with caution, particularly for antifungal agents commonly used in critically ill or immunocompromised patients. Mortality in the FAERS database may be strongly influenced by underlying disease severity, infection burden, comorbidities, and concomitant therapies rather than the direct effect of the suspected drug itself. As detailed clinical information is limited in FAERS, residual confounding by disease severity cannot be excluded. Therefore, death-related signals observed in this study should be interpreted as indicators of reporting patterns rather than direct evidence of drug-attributable mortality risk. Lastly, it is important to note that differences in approval dates and post-marketing duration among the three echinocandins may have influenced reporting patterns in spontaneous reporting systems. Caspofungin was approved earlier than micafungin and anidulafungin and therefore has a substantially longer reporting history. This may affect overall reporting frequencies because of the Weber effect, whereby adverse event reporting often peaks shortly after approval and subsequently declines over time (Li et al., 2026). In contrast, the relatively shorter post-marketing period of anidulafungin may partially contribute to its lower number of reports. Therefore, direct comparisons of reporting frequencies and signal strengths among the three agents should be interpreted cautiously.

## 5 Conclusion

By analyzing real-world data from the FAERS and VigiAccess databases, this study provides valuable insights into the safety profiles of three echinocandins. Several unlabeled AE signals were identified, which warrants further evaluation. Skin and subcutaneous tissue disorders associated with caspofungin and micafungin were significantly associated with hospitalization reporting, and supplementary PT-level analyses further identified several clinically relevant AEs associated with hospitalization. Higher disproportionality signals related to caspofungin and micafungin were observed in patients aged ≥65 years and females; however, these subgroup findings should be interpreted cautiously because exposure-adjusted prescribing data were unavailable. Hepatobiliary signals observed for anidulafungin also require cautious interpretation, as they are likely influenced by confounding by indication related to preferential use in patients with underlying hepatic dysfunction rather than direct drug toxicity. Furthermore, AEs associated with echinocandins were generally reported early after the initial treatment. Hence, maintaining appropriate vigilance against potential adverse effects contributes to enhanced patient safety, improved clinical decision-making, and increased effectiveness of drug therapies. Further clinical studies are needed to validate these findings.

## Data Availability

The FAERS data analyzed in this study are publicly available from the FDA website (https://fis.fda.gov/extensions/FPD-QDE-FAERS/FPD-QDE-FAERS.html) and were downloaded on July 20, 2025. VigiAccess data are publicly accessible through the WHO-Uppsala Monitoring Centre platform (http://www.vigiaccess.org/) and were retrieved on March 4, 2025. In addition, the analysis code used for data preprocessing, deduplication, and signal detection analyses is available from the corresponding author upon reasonable request.
