# Correction: Comparative real-world safety profiles of caspofungin, micafungin, and anidulafungin: a disproportionality analysis based on FAERS and VigiAccess databases

**DOI:** 10.3389/fphar.2026.1916009

**Published:** 2026-07-14

**Authors:** Jingxiu Chen, Chunhua Chen, Xiaobin Lin, Jiajia Yan, Yifan Zheng, Pan Chen, Jia Li

**Affiliations:** 1 Department of Pharmacy, The First Affiliated Hospital of Sun Yat-sen University, Guangzhou, China; 2 Department of Clinical Pharmacy Translational Science, University of Michigan College of Pharmacy, Ann Arbor, MI, United States; 3 Department of Pharmacy, Guangxi Hospital Division of the First Affiliated Hospital, Sun Yat-sen University, Nanning, China

**Keywords:** adverse event, anidulafungin, caspofungin, disproportionality analysis, micafungin

Affiliation 3 was erroneously duplicated from affiliation 2. The correct affiliation 3 is Department of Pharmacy, Guangxi Hospital Division of the First Affiliated Hospital, Sun Yat-sen University, Nanning, China.

The original version of this article has been updated.

