# Supplementary material for: Comparative real-world safety profiles of caspofungin, micafungin, and anidulafungin: a disproportionality analysis based on FAERS and VigiAccess databases
Source: Front Pharmacol. 2026 Jun 18;17:1840717. doi: 10.3389/fphar.2026.1840717 (PMC13323820; doi:10.3389/fphar.2026.1840717)
Supplement: Supplementary file 1 [file Supplementaryfile1.docx]

Supplementary Material

# Supplementary Figures and Tables

Disproportionation analysis is a data mining method, which is mainly used to evaluate the correlation between drugs and adverse reactions. The core principle is to use a 2 × 2 contingency table to compare the frequency of adverse events (AEs) observed in the exposed group and the non-exposed group, so as to quantify the association between drugs and adverse events. When the proportion of AEs in the exposed group exceeded that in the unexposed group, it was inferred that there was an association between drugs and specific AEs, indicating the presence of a disproportionation signal. After exceeding the threshold, the larger the signal value, the stronger the signal. In this study, we used four disproportional analysis methods: Reporting odds ratio (ROR), proportional reporting ratio (PRR), and Bayesian confidence propagation neural network (BCPNN). Specific data analysis methods are listed below:

Table S1. Two-by-two contingency table for disproportionality analysis.

|  | **Target AE** | **Other AE** | **Total** |
| --- | --- | --- | --- |
| Target drugs | a | b | a+b |
| Other drugs | c | d | c+d |
| Total | a+c | b+d | a+b+c+d |

Table S2. Disproportionality analysis methods and criteria for positive signal detection.

| Method | Calculation formula | ﻿Criteria |
| --- | --- | --- |
| ROR | $ROR=\frac{a / c}{b / d}$ | a ≥ 3  95%CI (lower limit) > 1 |
|  | $SE(lnROR)=\sqrt{\frac{1}{a}+\frac{1}{b}+\frac{1}{c}+\frac{1}{d}}$ |  |
|  | $95\%CI= e^{\ln\left( ROR \right)\pm1.96se}$ |  |
| PRR | $PRR=\frac{a / (a+b)}{c / (c+d)}$ | a ≥ 3  95%CI (lower limit) > 1 |
|  | $SE(lnPRR)=\sqrt{\frac{1}{a}-\frac{1}{a+b}+\frac{1}{c}-\frac{1}{c+d}}$ |  |
|  | $95\%CI= e^{\ln\left( PRR \right)\pm1.96se}$ |  |
|  | $\chi2 =\frac{{(ad-bc)}^{2}(a+b+c+d)}{( a+b)(a+c)(c+d)(b+d)}$ | a ≥ 3  PRR ≥ 2  $\chi2\geq4$ |
| BCPNN | IC=${log}_{2}\frac{p(x,y)}{p(x)p(y)}={log}_{2}\frac{a(a+b+c+d)}{(a+b)(a+c)}$ | IC025>0 |
|  | E(IC)=${log}_{2}\frac{(a+\gamma11)(a+b+c+d+\alpha)(a+b+c+d+\beta)}{（a+b+c+d+\gamma）(a+b+\alpha1)(a+c+\beta1)}$ |  |
|  | $V\left( IC \right)=\frac{1}{{(ln2)}^{2}}\{\left[ \frac{\left( a+b+c+d \right)-a+\gamma-\gamma11}{\left( a+\gamma11 \right)\left( 1+a+b+c+d+\gamma\right)} \right]+\left[ \frac{\left( a+b+c+d \right)-\left( a+b \right)+\alpha-\alpha1}{\left( a+b+\alpha1 \right)\left( 1+a+b+c+d+\alpha\right)} \right]+\left[ \frac{\left( a+b+c+d \right)-\left( a+c \right)+\beta-\beta1}{\left( a+c+\beta1 \right)\left( 1+a+b+c+d+\beta\right)} \right]\}$ |  |
|  | $\gamma=\gamma11\frac{(a+b+c+d+\alpha)(a+b+c+d+\beta)}{(a+b+\alpha1)(a+c+\beta1)}$ |  |
|  | *IC025=E(IC)-2*$\sqrt{V(IC)}$  $\alpha1=\beta1=1；\alpha=\beta=2；\gamma11=1$ |  |

Equation: a, number of reports containing both the target drug and target adverse drug reaction; b, number of reports containing other adverse drug reaction of the target drug; c, number of reports containing the target adverse drug reaction of other drugs; d, number of reports containing other drugs and other adverse drug reactions. ROR, reporting odds ratio; CI, confidence interval; PRR, proportional reporting ratio; χ^2^, chi-squared; BCPNN, bayesian confidence propagation neural network; IC, information component; IC025, the lower limit of 95%CI of the IC. 95%CI, 95% confidence interval; E (IC), the IC expectations; V (IC), the variance of IC;

Figure S1 Number of annual reports of echinocandin-related adverse events from the FAERS database.

Table S3 Drug-unrelated AE signals excluded from the FAERS database.

| **SOC** | | **PT** | | **N** | | **ROR (95% CI)** | |
| --- | --- | --- | --- | --- | --- | --- | --- |
| **Caspofungin** | | | | | | | |
| Investigations | | Antimicrobial susceptibility test resistant | | 4 | | 140.24(52.24,376.50) | |
| General disorders and administration site conditions | | Therapy responder | | 6 | | 124.87(55.78,279.53) | |
| Investigations | | Fungal test positive | | 3 | | 60.41(19.41,188.05) | |
| General disorders and administration site conditions | | Drug resistance | | 53 | | 21.97(16.76,28.80) | |
| General disorders and administration site conditions | | Multiple-drug resistance | | 3 | | 10.58(3.41,32.83) | |
| General disorders and administration site conditions | | Therapy non-responder | | 40 | | 7.62(5.58,10.40) | |
| General disorders and administration site conditions | | Treatment failure | | 23 | | 3.10(2.05,4.66) | |
| General disorders and administration site conditions | | Adverse event | | 18 | | 2.03(1.28,3.23) | |
| Infections and infestations | | Scopulariopsis infection | | 3 | | 788.73(242.82,2561.91) | |
| Infections and infestations | | Upper respiratory fungal infection | | 8 | | 464.92(228.52,945.85) | |
| Infections and infestations | | Hepatosplenic candidiasis | | 4 | | 289.05(106.85,781.93) | |
| Infections and infestations | | Hepatic infection fungal | | 3 | | 273.02(86.61,860.61) | |
| Infections and infestations | | Systemic mycosis | | 24 | | 231.19(154.09,346.88) | |
| Injury, poisoning and procedural complications | | Mechanical ventilation complication | | 3 | | 207.26(66.01,650.78) | |
| Infections and infestations | | Geotrichum infection | | 4 | | 201.41(74.79,542.42) | |
| Infections and infestations | | Systemic candida | | 43 | | 199.48(147.32,270.10) | |
| Infections and infestations | | Trichosporon infection | | 7 | | 193.29(91.42,408.68) | |
| Infections and infestations | | Cerebral aspergillosis | | 10 | | 181.53(97.05,339.56) | |
| Infections and infestations | | Fungaemia | | 24 | | 168.60(112.52,252.64) | |
| Infections and infestations | | Fungal endocarditis | | 4 | | 146.76(54.65,394.15) | |
| Infections and infestations | | Fungal sepsis | | 11 | | 137.72(75.90,249.88) | |
| Infections and infestations | | Cerebral fungal infection | | 3 | | 117.33(37.57,366.46) | |
| Infections and infestations | | Candida sepsis | | 9 | | 104.62(54.21,201.90) | |
| Infections and infestations | | Fusarium infection | | 6 | | 97.46(43.59,217.91) | |
| Infections and infestations | | Aspergillus infection | | 64 | | 88.97(69.47,113.95) | |
| Infections and infestations | | Bronchopulmonary aspergillosis | | 57 | | 79.16(60.92,102.86) | |
| Infections and infestations | | Respiratory tract infection fungal | | 4 | | 74.39(27.80,199.04) | |
| Infections and infestations | | Pathogen resistance | | 59 | | 68.95(53.30,89.18) | |
| Infections and infestations | | Renal abscess | | 5 | | 67.34(27.93,162.35) | |
| Infections and infestations | | Mucormycosis | | 19 | | 65.83(41.89,103.44) | |
| Infections and infestations | | Septic embolus | | 5 | | 61.40(25.47,147.99) | |
| Infections and infestations | | Enterococcal bacteraemia | | 5 | | 51.12(21.22,123.16) | |
| Infections and infestations | | Stenotrophomonas infection | | 6 | | 49.58(22.22,110.63) | |
| Infections and infestations | | Scedosporium infection | | 3 | | 49.21(15.82,153.07) | |
| Injury, poisoning and procedural complications | | Labelled drug-drug interaction issue | | 5 | | 48.55(20.16,116.96) | |
| Neoplasms benign, malignant and unspecified (incl cysts and polyps) | | Leukaemia recurrent | | 7 | | 41.72(19.85,87.70) | |
| Infections and infestations | | Adenovirus infection | | 8 | | 30.40(15.18,60.88) | |
| Infections and infestations | | Pneumonia pneumococcal | | 4 | | 28.30(10.60,75.54) | |
| Surgical and medical procedures | | Mechanical ventilation | | 5 | | 26.46(11.00,63.67) | |
| Injury, poisoning and procedural complications | | Transplant failure | | 6 | | 26.01(11.67,57.99) | |
| Infections and infestations | | Endocarditis | | 12 | | 24.27(13.77,42.80) | |
| Infections and infestations | | Pneumonia fungal | | 10 | | 23.18(12.46,43.13) | |
| Infections and infestations | | Candida infection | | 42 | | 21.61(15.94,29.28) | |
| Neoplasms benign, malignant and unspecified (incl cysts and polyps) | | Acute lymphocytic leukaemia | | 6 | | 21.59(9.69,48.13) | |
| Infections and infestations | | Septic shock | | 85 | | 21.14(17.06,26.19) | |
| Infections and infestations | | Abdominal infection | | 5 | | 20.62(8.57,49.60) | |
| Infections and infestations | | Liver abscess | | 6 | | 19.73(8.85,43.98) | |
| Neoplasms benign, malignant and unspecified (incl cysts and polyps) | | Acute myeloid leukaemia | | 28 | | 18.90(13.03,27.40) | |
| Infections and infestations | | Pseudomembranous colitis | | 4 | | 18.09(6.78,48.26) | |
| Infections and infestations | | Fungal infection | | 56 | | 17.62(13.54,22.93) | |
| Infections and infestations | | Enterococcal infection | | 8 | | 17.44(8.71,34.90) | |
| Infections and infestations | | Cytomegalovirus chorioretinitis | | 3 | | 16.80(5.41,52.16) | |
| Surgical and medical procedures | | Bone marrow transplant | | 3 | | 16.74(5.39,51.98) | |
| Infections and infestations | | Pneumonia bacterial | | 14 | | 16.71(9.88,28.24) | |
| Infections and infestations | | Pneumonia pseudomonal | | 3 | | 15.05(4.85,46.73) | |
| Infections and infestations | | Staphylococcal bacteraemia | | 6 | | 15.01(6.74,33.45) | |
| Infections and infestations | | Staphylococcal sepsis | | 7 | | 14.23(6.78,29.89) | |
| Infections and infestations | | Bacterial sepsis | | 4 | | 12.75(4.78,34.00) | |
| Neoplasms benign, malignant and unspecified (incl cysts and polyps) | | Haematological malignancy | | 3 | | 10.73(3.46,33.30) | |
| Infections and infestations | | Oesophageal candidiasis | | 4 | | 10.71(4.02,28.57) | |
| Infections and infestations | | Klebsiella infection | | 5 | | 10.65(4.43,25.60) | |
| Infections and infestations | | Cytomegalovirus infection | | 15 | | 9.44(5.69,15.68) | |
| Neoplasms benign, malignant and unspecified (incl cysts and polyps) | | B-cell lymphoma | | 3 | | 8.94(2.88,27.73) | |
| Infections and infestations | | Bacterial infection | | 14 | | 8.05(4.76,13.60) | |
| Neoplasms benign, malignant and unspecified (incl cysts and polyps) | | Lymphoma | | 11 | | 7.32(4.05,13.23) | |
| Infections and infestations | | Bacteraemia | | 8 | | 7.27(3.64,14.56) | |
| Infections and infestations | | Sepsis | | 76 | | 7.13(5.69,8.94) | |
| Infections and infestations | | Pseudomonas infection | | 5 | | 6.31(2.63,15.18) | |
| Infections and infestations | | Encephalitis | | 4 | | 6.15(2.31,16.40) | |
| Injury, poisoning and procedural complications | | Product use in unapproved indication | | 128 | | 5.95(5.00,7.09) | |
| Infections and infestations | | Clostridium difficile colitis | | 6 | | 5.90(2.65,13.15) | |
| Infections and infestations | | Peritonitis | | 12 | | 5.73(3.25,10.10) | |
| Neoplasms benign, malignant and unspecified (incl cysts and polyps) | | Leukaemia | | 5 | | 5.51(2.29,13.24) | |
| Infections and infestations | | Respiratory syncytial virus infection | | 4 | | 5.24(1.96,13.96) | |
| Infections and infestations | | Escherichia infection | | 4 | | 5.22(1.96,13.93) | |
| Neoplasms benign, malignant and unspecified (incl cysts and polyps) | | Myelodysplastic syndrome | | 7 | | 4.95(2.36,10.38) | |
| Infections and infestations | | Staphylococcal infection | | 15 | | 4.62(2.78,7.67) | |
| Infections and infestations | | Device related infection | | 7 | | 4.32(2.06,9.06) | |
| Infections and infestations | | Pneumocystis jirovecii pneumonia | | 5 | | 4.21(1.75,10.13) | |
| Infections and infestations | | Pneumonia | | 76 | | 2.33(1.86,2.92) | |
| Injury, poisoning and procedural complications | | Medication error | | 12 | | 2.26(1.29,3.99) | |
| Investigations | | Drug level increased | | 5 | | 3.09(1.29,7.43) | |
| General disorders and administration site conditions | | Drug ineffective | | 524 | | 4.45(4.07,4.87) | |
| Investigations | | Blood culture positive | | 6 | | 19.47(8.74,43.39) | |
| **Micafungin** | | | | | | | |
| Injury, poisoning and procedural complications | | Off label use | | 538 | | 7.51(6.87,8.20) | |
| Injury, poisoning and procedural complications | | Product use issue | | 90 | | 5.33(4.33,6.57) | |
| Infections and infestations | | Septic shock | | 65 | | 16.35(12.80,20.89) | |
| Infections and infestations | | Sepsis | | 57 | | 5.41(4.17,7.03) | |
| Infections and infestations | | Bronchopulmonary aspergillosis | | 51 | | 71.79(54.44,94.67) | |
| Injury, poisoning and procedural complications | | Product use in unapproved indication | | 51 | | 2.38(1.80,3.13) | |
| Infections and infestations | | Infection | | 47 | | 3.55(2.66,4.73) | |
| Infections and infestations | | Candida infection | | 47 | | 24.58(18.44,32.76) | |
| Infections and infestations | | Aspergillus infection | | 34 | | 47.55(33.91,66.66) | |
| Injury, poisoning and procedural complications | | Product storage error | | 29 | | 3.22(2.23,4.64) | |
| Infections and infestations | | Systemic candida | | 28 | | 130.61(89.87,189.80) | |
| Infections and infestations | | Fungal infection | | 27 | | 8.58(5.88,12.52) | |
| Infections and infestations | | Mucormycosis | | 25 | | 88.24(59.46,130.93) | |
| Infections and infestations | | Trichosporon infection | | 23 | | 678.25(444.04,1035.99) | |
| Infections and infestations | | Systemic mycosis | | 18 | | 174.82(109.61,278.83) | |
| Infections and infestations | | Candida sepsis | | 16 | | 190.72(116.20,313.03) | |
| Infections and infestations | | Fungaemia | | 14 | | 98.97(58.42,167.66) | |
| Infections and infestations | | Cytomegalovirus infection | | 13 | | 8.31(4.82,14.32) | |
| Infections and infestations | | Staphylococcal infection | | 12 | | 3.75(2.13,6.61) | |
| Infections and infestations | | Bacterial infection | | 12 | | 7.00(3.97,12.34) | |
| Infections and infestations | | Device related infection | | 11 | | 6.89(3.81,12.45) | |
| Infections and infestations | | Peritonitis | | 10 | | 4.85(2.61,9.01) | |
| Infections and infestations | | Pneumonia fungal | | 10 | | 23.53(12.65,43.80) | |
| Surgical and medical procedures | | Haemodialysis | | 9 | | 10.38(5.40,19.97) | |
| Neoplasms benign, malignant and unspecified (incl cysts and polyps) | | Leukaemia | | 9 | | 10.08(5.24,19.39) | |
| Neoplasms benign, malignant and unspecified (incl cysts and polyps) | | Acute myeloid leukaemia | | 8 | | 5.46(2.73,10.92) | |
| Infections and infestations | | Pneumonia aspiration | | 8 | | 3.43(1.71,6.86) | |
| Infections and infestations | | Pseudomonas infection | | 8 | | 10.27(5.13,20.54) | |
| Infections and infestations | | Pneumocystis jirovecii pneumonia | | 8 | | 6.85(3.42,13.71) | |
| Injury, poisoning and procedural complications | | Prescribed overdose | | 7 | | 3.92(1.87,8.22) | |
| Infections and infestations | | Brain abscess | | 6 | | 25.07(11.25,55.89) | |
| Infections and infestations | | Eye infection fungal | | 6 | | 172.21(76.78,386.22) | |
| Infections and infestations | | Fusarium infection | | 6 | | 98.95(44.25,221.26) | |
| Infections and infestations | | Infectious pleural effusion | | 6 | | 40.68(18.24,90.75) | |
| Infections and infestations | | Bacteraemia | | 5 | | 4.61(1.92,11.09) | |
| Infections and infestations | | Endocarditis | | 5 | | 10.24(4.26,24.63) | |
| Infections and infestations | | Nosocomial infection | | 5 | | 23.20(9.64,55.81) | |
| Infections and infestations | | Acinetobacter infection | | 5 | | 49.25(20.44,118.63) | |
| Infections and infestations | | Fungal sepsis | | 5 | | 63.00(26.14,151.84) | |
| Infections and infestations | | Pseudomonal sepsis | | 5 | | 30.62(12.72,73.68) | |
| Infections and infestations | | Candida endophthalmitis | | 5 | | 480.67(195.71,1180.54) | |
| Infections and infestations | | Enterococcal infection | | 5 | | 11.05(4.60,26.58) | |
| Infections and infestations | | Klebsiella infection | | 5 | | 10.81(4.50,26.00) | |
| Infections and infestations | | Clostridium difficile colitis | | 4 | | 3.99(1.50,10.65) | |
| Infections and infestations | | Liver abscess | | 4 | | 13.34(5.00,35.59) | |
| Infections and infestations | | Meningitis | | 4 | | 6.38(2.39,17.02) | |
| Infections and infestations | | Pseudomembranous colitis | | 4 | | 18.37(6.89,49.00) | |
| Infections and infestations | | Enterococcal sepsis | | 4 | | 51.19(19.16,136.81) | |
| Infections and infestations | | Herpes simplex oesophagitis | | 4 | | 252.94(93.69,682.86) | |
| Infections and infestations | | Disseminated cryptococcosis | | 3 | | 46.65(15.00,145.09) | |
| Infections and infestations | | Enterococcal bacteraemia | | 3 | | 31.07(10.00,96.53) | |
| Infections and infestations | | Fungal endocarditis | | 3 | | 111.31(35.66,347.51) | |
| Infections and infestations | | Infection in an immunocompromised host | | 3 | | 47.65(15.32,148.21) | |
| Infections and infestations | | Meningitis fungal | | 3 | | 138.61(44.33,433.40) | |
| Infections and infestations | | Oesophageal candidiasis | | 3 | | 8.16(2.63,25.31) | |
| Infections and infestations | | Pneumonia klebsiella | | 3 | | 17.29(5.57,53.69) | |
| Infections and infestations | | Pneumonia pseudomonal | | 3 | | 15.29(4.92,47.45) | |
| Infections and infestations | | Staphylococcal bacteraemia | | 3 | | 7.61(2.45,23.62) | |
| Infections and infestations | | Klebsiella sepsis | | 3 | | 28.49(9.17,88.50) | |
| Infections and infestations | | Abdominal sepsis | | 3 | | 36.49(11.74,113.43) | |
| Infections and infestations | | Sinusitis fungal | | 3 | | 43.22(13.90,134.40) | |
| Infections and infestations | | Oral fungal infection | | 3 | | 11.35(3.66,35.23) | |
| Surgical and medical procedures | | Bone marrow transplant | | 3 | | 17.00(5.48,52.77) | |
| Surgical and medical procedures | | Stem cell transplant | | 3 | | 13.13(4.23,40.75) | |
| Infections and infestations | | Cutaneous mucormycosis | | 3 | | 220.08(70.05,691.43) | |
| Congenital, familial and genetic disorders | | Congenital aplasia | | 3 | | 13.21(4.25,40.99) | |
| General disorders and administration site conditions | | Drug resistance | | 26 | | 10.88(7.40,16.00) | |
| General disorders and administration site conditions | | Drug ineffective for unapproved indication | | 24 | | 4.65(3.12,6.95) | |
| Investigations | | Blood culture positive | | 6 | | 19.77(8.87,44.06) | |
| General disorders and administration site conditions | | Injection site extravasation | | 5 | | 4.00(1.67,9.63) | |
| Investigations | | Candida test positive | | 5 | | 115.27(47.71,278.50) | |
| Investigations | | Laboratory test interference | | 3 | | 38.08(12.25,118.39) | |
| Investigations | | Blood beta-D-glucan increased | | 8 | | 282.89(139.98,571.70) | |
| Investigations | | Sputum culture positive | | 4 | | 48.00(17.96,128.24) | |
| **Anidulafungin** | | | | | | | |
| Injury, poisoning and procedural complications | | Off label use | | 31 | | 2.25(1.58,3.22) | |
| Infections and infestations | | Septic shock | | 19 | | 26.77(17.01,42.15) | |
| Infections and infestations | | Sepsis | | 11 | | 5.82(3.21,10.54) | |
| Infections and infestations | | Candida infection | | 9 | | 26.17(13.58,50.44) | |
| Infections and infestations | | Fungal infection | | 6 | | 10.62(4.76,23.69) | |
| Infections and infestations | | Systemic candida | | 5 | | 128.44(53.29,309.55) | |
| Infections and infestations | | Fungal peritonitis | | 5 | | 187.77(77.87,452.76) | |
| Infections and infestations | | Bacterial infection | | 4 | | 13.01(4.87,34.74) | |
| Infections and infestations | | Pathogen resistance | | 3 | | 19.55(6.29,60.72) | |
| General disorders and administration site conditions | | Drug ineffective for unapproved indication | | 9 | | 9.76(5.06,18.81) | |

SOC, system organ class; PT, preferred term; ROR, reporting odds ratio; CI, confidence interval.

Table S4 Signal strength of adverse events associated with caspofungin at the PT level ranked by ROR in FAERS.

| SOC | PT | N | ROR (95% CI) | PRR (χ^2^) | IC (IC025) |
| --- | --- | --- | --- | --- | --- |
| Skin and subcutaneous tissue disorders | Cutaneous calcification | 5 | 361.37 (147.88,883.04) | 361.07 (1729.35) | 8.44 (1.36) |
| Nervous system disorders | Frontotemporal dementia | 3 | 73.75 (23.67,229.75) | 73.71 (213.52) | 6.19 (0.49) |
| Immune system disorders | Engraftment syndrome | 3 | 55.03 (17.68,171.23) | 55.00 (158.14) | 5.77 (0.47) |
| Immune system disorders | Graft versus host disease | 28 | 39.67 (27.35,57.56) | 39.49 (1046.27) | 5.30 (3.54) |
| General disorders and administration site conditions | Mucosal haemorrhage | 4 | 35.72 (13.38,95.39) | 35.70 (134.40) | 5.15 (0.87) |
| Blood and lymphatic system disorders | Hypofibrinogenaemia | 3 | 32.83 (10.56,102.01) | 32.81 (92.20) | 5.03 (0.43) |
| General disorders and administration site conditions | Organ failure | 11 | 27.67 (15.30,50.03) | 27.62 (281.38) | 4.78 (2.26) |
| General disorders and administration site conditions | Multiple organ dysfunction syndrome | 111 | 26.24 (21.74,31.66) | 25.77 (2636.97) | 4.68 (4.12) |
| Renal and urinary disorders | Renal tubular disorder | 7 | 24.88 (11.84,52.25) | 24.85 (159.81) | 4.63 (1.62) |
| Hepatobiliary disorders | Mixed liver injury | 6 | 24.51 (11.00,54.64) | 24.49 (134.82) | 4.61 (1.40) |
| Hepatobiliary disorders | Cholestasis | 44 | 24.35 (18.10,32.77) | 24.18 (975.62) | 4.59 (3.56) |
| General disorders and administration site conditions | Death neonatal | 3 | 23.14 (7.45,71.87) | 23.13 (63.36) | 4.53 (0.38) |
| Immune system disorders | Acute graft versus host disease | 10 | 22.37 (12.02,41.63) | 22.34 (203.36) | 4.48 (2.05) |
| Metabolism and nutrition disorders | Cell death | 4 | 21.13 (7.92,56.38) | 21.12 (76.49) | 4.40 (0.78) |
| Cardiac disorders | Cardiopulmonary failure | 8 | 20.49 (10.23,41.01) | 20.46 (147.76) | 4.35 (1.73) |
| Vascular disorders | Venoocclusive disease | 5 | 20.26 (8.42,48.73) | 20.24 (91.25) | 4.34 (1.09) |
| Skin and subcutaneous tissue disorders | Toxic skin eruption | 19 | 19.45 (12.39,30.54) | 19.40 (330.87) | 4.27 (2.69) |
| Investigations | Blood alkaline phosphatase increased | 47 | 19.12 (14.34,25.48) | 18.97 (798.91) | 4.24 (3.37) |
| Investigations | Bilirubin conjugated increased | 4 | 17.66 (6.62,47.11) | 17.65 (62.71) | 4.14 (0.73) |
| Immune system disorders | Haemophagocytic lymphohistiocytosis | 16 | 17.60 (10.77,28.76) | 17.56 (249.37) | 4.13 (2.45) |
| Hepatobiliary disorders | Jaundice cholestatic | 6 | 17.58 (7.89,39.17) | 17.56 (93.54) | 4.13 (1.29) |
| Investigations | Blood lactate dehydrogenase increased | 28 | 17.55 (12.11,25.45) | 17.48 (434.24) | 4.12 (2.94) |
| Hepatobiliary disorders | Venoocclusive liver disease | 8 | 17.19 (8.59,34.41) | 17.17 (121.60) | 4.10 (1.65) |
| Metabolism and nutrition disorders | Hypoproteinaemia | 3 | 17.05 (5.49,52.94) | 17.04 (45.23) | 4.09 (0.32) |
| Skin and subcutaneous tissue disorders | Drug reaction with eosinophilia and systemic symptoms | 45 | 16.80 (12.53,22.53) | 16.68 (662.53) | 4.06 (3.21) |
| Respiratory, thoracic and mediastinal disorders | Pulmonary haemorrhage | 13 | 16.76 (9.72,28.90) | 16.73 (191.92) | 4.06 (2.20) |
| Blood and lymphatic system disorders | Eosinophilia | 27 | 16.45 (11.27,24.02) | 16.38 (389.38) | 4.03 (2.85) |
| Hepatobiliary disorders | Cholestatic liver injury | 3 | 16.22 (5.23,50.37) | 16.22 (42.76) | 4.02 (0.31) |
| Investigations | Neutrophil count increased | 16 | 15.84 (9.69,25.88) | 15.80 (221.42) | 3.98 (2.38) |
| Skin and subcutaneous tissue disorders | Dermatitis exfoliative | 8 | 15.67 (7.83,31.36) | 15.65 (109.51) | 3.97 (1.61) |
| Blood and lymphatic system disorders | Disseminated intravascular coagulation | 20 | 14.42 (9.29,22.37) | 14.37 (248.50) | 3.84 (2.50) |
| Hepatobiliary disorders | Hepatitis cholestatic | 8 | 14.25 (7.12,28.51) | 14.23 (98.25) | 3.83 (1.56) |
| Nervous system disorders | Paraplegia | 4 | 14.15 (5.31,37.75) | 14.15 (48.80) | 3.82 (0.67) |
| Respiratory, thoracic and mediastinal disorders | Acute respiratory distress syndrome | 24 | 14.13 (9.46,21.10) | 14.07 (291.11) | 3.81 (2.63) |
| Investigations | Gamma-glutamyltransferase increased | 31 | 14.12 (9.92,20.10) | 14.05 (375.43) | 3.81 (2.81) |
| Blood and lymphatic system disorders | Agranulocytosis | 24 | 13.96 (9.35,20.85) | 13.91 (287.21) | 3.80 (2.62) |
| Hepatobiliary disorders | Hepatic necrosis | 5 | 13.29 (5.53,31.96) | 13.28 (56.70) | 3.73 (0.94) |
| Metabolism and nutrition disorders | Hypernatraemia | 6 | 12.64 (5.67,28.15) | 12.62 (64.14) | 3.66 (1.15) |
| Hepatobiliary disorders | Hyperbilirubinaemia | 12 | 12.35 (7.01,21.77) | 12.33 (124.79) | 3.62 (1.92) |
| Hepatobiliary disorders | Hepatocellular injury | 20 | 12.17 (7.85,18.89) | 12.14 (204.18) | 3.60 (2.36) |
| Hepatobiliary disorders | Hepatic cytolysis | 15 | 11.68 (7.03,19.39) | 11.65 (145.92) | 3.54 (2.08) |
| Respiratory, thoracic and mediastinal disorders | Pulmonary alveolar haemorrhage | 6 | 11.55 (5.18,25.73) | 11.54 (57.69) | 3.53 (1.11) |
| Skin and subcutaneous tissue disorders | Rash morbilliform | 3 | 11.41 (3.68,35.40) | 11.40 (28.44) | 3.51 (0.22) |
| General disorders and administration site conditions | Hyperpyrexia | 4 | 10.98 (4.12,29.29) | 10.98 (36.24) | 3.45 (0.58) |
| Skin and subcutaneous tissue disorders | Rash maculo-papular | 23 | 10.98 (7.29,16.54) | 10.94 (207.60) | 3.45 (2.36) |
| Respiratory, thoracic and mediastinal disorders | Acute pulmonary oedema | 6 | 10.89 (4.89,24.27) | 10.88 (53.79) | 3.44 (1.08) |
| Skin and subcutaneous tissue disorders | Toxic epidermal necrolysis | 15 | 10.54 (6.35,17.49) | 10.51 (128.99) | 3.39 (2.00) |
| Respiratory, thoracic and mediastinal disorders | Hypercapnia | 3 | 10.47 (3.37,32.49) | 10.46 (25.65) | 3.39 (0.19) |
| Blood and lymphatic system disorders | Bone marrow disorder | 3 | 10.33 (3.33,32.07) | 10.33 (25.25) | 3.37 (0.19) |
| Hepatobiliary disorders | Hepatitis fulminant | 3 | 10.31 (3.32,32.01) | 10.31 (25.19) | 3.36 (0.19) |
| Investigations | Blood bilirubin increased | 27 | 10.21 (6.99,14.90) | 10.17 (223.01) | 3.34 (2.39) |
| Gastrointestinal disorders | Enterocolitis | 5 | 9.75 (4.06,23.45) | 9.74 (39.20) | 3.28 (0.81) |
| Hepatobiliary disorders | Hepatic failure | 28 | 9.59 (6.61,13.90) | 9.55 (214.09) | 3.25 (2.34) |
| Investigations | C-reactive protein increased | 32 | 9.43 (6.66,13.35) | 9.39 (239.66) | 3.23 (2.40) |
| Investigations | Transaminases increased | 20 | 9.37 (6.04,14.54) | 9.35 (148.97) | 3.22 (2.11) |
| Hepatobiliary disorders | Hepatotoxicity | 19 | 9.16 (5.84,14.38) | 9.14 (137.60) | 3.19 (2.05) |
| Cardiac disorders | Myocardial injury | 3 | 9.16 (2.95,28.41) | 9.15 (21.76) | 3.19 (0.15) |
| Hepatobiliary disorders | Hepatic function abnormal | 31 | 8.94 (6.28,12.73) | 8.90 (217.36) | 3.15 (2.32) |
| Renal and urinary disorders | Nephropathy toxic | 9 | 8.78 (4.57,16.89) | 8.77 (61.92) | 3.13 (1.39) |
| Skin and subcutaneous tissue disorders | Cutaneous vasculitis | 4 | 8.58 (3.22,22.88) | 8.58 (26.75) | 3.10 (0.48) |
| Respiratory, thoracic and mediastinal disorders | Respiratory failure | 60 | 8.52 (6.61,10.99) | 8.45 (394.16) | 3.08 (2.54) |
| Hepatobiliary disorders | Drug-induced liver injury | 22 | 8.26 (5.44,12.56) | 8.24 (139.84) | 3.04 (2.04) |
| Cardiac disorders | Cardiac failure acute | 5 | 7.98 (3.32,19.19) | 7.98 (30.48) | 2.99 (0.70) |
| Investigations | Aspartate aminotransferase increased | 41 | 7.96 (5.86,10.83) | 7.92 (247.73) | 2.98 (2.32) |
| Psychiatric disorders | Dysphoria | 4 | 7.95 (2.98,21.20) | 7.95 (24.27) | 2.99 (0.44) |
| Hepatobiliary disorders | Liver injury | 16 | 7.79 (4.77,12.72) | 7.77 (94.32) | 2.96 (1.77) |
| Renal and urinary disorders | Cystitis haemorrhagic | 3 | 7.75 (2.50,24.04) | 7.75 (17.61) | 2.95 (0.08) |
| Investigations | Alanine aminotransferase increased | 45 | 7.58 (5.65,10.16) | 7.53 (254.68) | 2.91 (2.29) |
| Respiratory, thoracic and mediastinal disorders | Acute respiratory failure | 13 | 7.21 (4.18,12.42) | 7.19 (69.29) | 2.85 (1.55) |
| Hepatobiliary disorders | Acute hepatic failure | 9 | 7.16 (3.72,13.78) | 7.15 (47.62) | 2.84 (1.23) |
| Investigations | White blood cell count increased | 27 | 7.12 (4.88,10.39) | 7.09 (141.20) | 2.82 (1.99) |
| Nervous system disorders | Encephalopathy | 16 | 6.87 (4.20,11.22) | 6.85 (79.93) | 2.78 (1.65) |
| Skin and subcutaneous tissue disorders | Acute generalised exanthematous pustulosis | 5 | 6.79 (2.82,16.32) | 6.78 (24.63) | 2.76 (0.61) |
| Metabolism and nutrition disorders | Acidosis | 5 | 6.71 (2.79,16.13) | 6.70 (24.25) | 2.74 (0.60) |
| Metabolism and nutrition disorders | Malnutrition | 7 | 6.66 (3.17,13.97) | 6.65 (33.58) | 2.73 (0.94) |
| Investigations | Prothrombin time prolonged | 4 | 6.41 (2.40,17.10) | 6.41 (18.25) | 2.68 (0.33) |
| Respiratory, thoracic and mediastinal disorders | Respiratory distress | 17 | 6.35 (3.95,10.23) | 6.34 (76.40) | 2.66 (1.61) |
| Metabolism and nutrition disorders | Hypokalaemia | 27 | 6.16 (4.22,8.99) | 6.14 (116.15) | 2.62 (1.83) |
| Nervous system disorders | Hemiplegia | 5 | 6.12 (2.54,14.70) | 6.11 (21.36) | 2.61 (0.54) |
| Metabolism and nutrition disorders | Hypercalcaemia | 7 | 5.93 (2.83,12.46) | 5.93 (28.67) | 2.57 (0.85) |
| Hepatobiliary disorders | Hepatitis | 14 | 5.73 (3.39,9.68) | 5.72 (54.49) | 2.51 (1.37) |
| Respiratory, thoracic and mediastinal disorders | Lung infiltration | 5 | 5.52 (2.30,13.28) | 5.52 (18.50) | 2.46 (0.48) |
| Skin and subcutaneous tissue disorders | Stevens-Johnson syndrome | 12 | 5.52 (3.13,9.72) | 5.51 (44.25) | 2.46 (1.23) |
| Blood and lymphatic system disorders | Pancytopenia | 29 | 5.46 (3.79,7.87) | 5.44 (105.21) | 2.44 (1.72) |
| Vascular disorders | Circulatory collapse | 9 | 5.34 (2.78,10.28) | 5.34 (31.72) | 2.42 (0.98) |
| Investigations | Liver function test abnormal | 15 | 5.15 (3.10,8.55) | 5.14 (50.06) | 2.36 (1.31) |
| Cardiac disorders | Atrial flutter | 4 | 5.08 (1.91,13.55) | 5.08 (13.10) | 2.34 (0.19) |
| Hepatobiliary disorders | Liver disorder | 21 | 4.99 (3.25,7.66) | 4.97 (66.69) | 2.31 (1.46) |
| Metabolism and nutrition disorders | Tumour lysis syndrome | 4 | 4.93 (1.85,13.16) | 4.93 (12.53) | 2.30 (0.17) |
| Hepatobiliary disorders | Jaundice | 13 | 4.88 (2.83,8.41) | 4.87 (39.99) | 2.28 (1.16) |
| Respiratory, thoracic and mediastinal disorders | Tachypnoea | 6 | 4.66 (2.09,10.38) | 4.66 (17.23) | 2.22 (0.52) |
| Vascular disorders | Shock | 10 | 4.65 (2.50,8.65) | 4.65 (28.60) | 2.22 (0.93) |
| Skin and subcutaneous tissue disorders | Purpura | 4 | 4.62 (1.73,12.33) | 4.62 (11.35) | 2.21 (0.13) |
| Respiratory, thoracic and mediastinal disorders | Pneumothorax | 7 | 4.33 (2.07,9.10) | 4.33 (17.93) | 2.11 (0.59) |
| Cardiac disorders | Sinus bradycardia | 4 | 4.22 (1.58,11.25) | 4.22 (9.81) | 2.08 (0.07) |
| Gastrointestinal disorders | Ascites | 12 | 4.20 (2.39,7.41) | 4.20 (29.22) | 2.07 (0.95) |
| Blood and lymphatic system disorders | Bone marrow failure | 8 | 3.91 (1.96,7.83) | 3.91 (17.31) | 1.97 (0.60) |
| Metabolism and nutrition disorders | Hypomagnesaemia | 5 | 3.81 (1.58,9.16) | 3.81 (10.35) | 1.93 (0.20) |
| Respiratory, thoracic and mediastinal disorders | Interstitial lung disease | 17 | 3.76 (2.34,6.05) | 3.75 (34.33) | 1.91 (1.02) |
| Immune system disorders | Anaphylactic shock | 9 | 3.74 (1.95,7.20) | 3.74 (18.05) | 1.90 (0.64) |
| Blood and lymphatic system disorders | Febrile neutropenia | 23 | 3.69 (2.45,5.55) | 3.68 (44.83) | 1.88 (1.13) |
| Psychiatric disorders | Delirium | 12 | 3.67 (2.08,6.47) | 3.66 (23.25) | 1.87 (0.80) |
| General disorders and administration site conditions | Face oedema | 6 | 3.61 (1.62,8.04) | 3.61 (11.30) | 1.85 (0.30) |
| Renal and urinary disorders | Renal failure | 46 | 3.47 (2.60,4.64) | 3.45 (80.34) | 1.79 (1.29) |
| Investigations | Neutrophil count decreased | 12 | 3.16 (1.79,5.57) | 3.15 (17.66) | 1.66 (0.63) |
| Investigations | Electrocardiogram QT prolonged | 11 | 3.15 (1.74,5.70) | 3.15 (16.14) | 1.65 (0.58) |
| Cardiac disorders | Cardio-respiratory arrest | 13 | 3.10 (1.80,5.34) | 3.09 (18.43) | 1.63 (0.66) |
| Skin and subcutaneous tissue disorders | Drug eruption | 5 | 3.04 (1.26,7.30) | 3.03 (6.82) | 1.60 (0.00) |
| Investigations | Hepatic enzyme increased | 18 | 2.88 (1.82,4.58) | 2.88 (22.07) | 1.52 (0.73) |
| Respiratory, thoracic and mediastinal disorders | Respiratory disorder | 8 | 2.79 (1.39,5.57) | 2.78 (9.14) | 1.48 (0.25) |
| Respiratory, thoracic and mediastinal disorders | Haemoptysis | 7 | 2.57 (1.23,5.40) | 2.57 (6.73) | 1.36 (0.08) |
| Blood and lymphatic system disorders | Leukopenia | 12 | 2.52 (1.43,4.43) | 2.51 (10.94) | 1.33 (0.37) |
| Nervous system disorders | Epilepsy | 7 | 2.43 (1.16,5.10) | 2.43 (5.89) | 1.28 (0.02) |
| Gastrointestinal disorders | Pancreatitis | 12 | 2.36 (1.34,4.15) | 2.35 (9.34) | 1.23 (0.29) |
| Investigations | Blood creatinine increased | 15 | 2.35 (1.41,3.90) | 2.34 (11.56) | 1.23 (0.39) |
| Nervous system disorders | Cerebral haemorrhage | 8 | 2.30 (1.15,4.60) | 2.30 (5.87) | 1.20 (0.04) |
| Blood and lymphatic system disorders | Thrombocytopenia | 24 | 2.26 (1.51,3.37) | 2.25 (16.76) | 1.17 (0.52) |
| Respiratory, thoracic and mediastinal disorders | Pleural effusion | 13 | 2.18 (1.27,3.76) | 2.18 (8.31) | 1.12 (0.24) |
| Renal and urinary disorders | Renal impairment | 17 | 2.14 (1.33,3.45) | 2.14 (10.35) | 1.10 (0.33) |
| Cardiac disorders | Cardiac failure | 16 | 2.06 (1.26,3.37) | 2.06 (8.74) | 1.04 (0.25) |
| Renal and urinary disorders | Acute kidney injury | 38 | 2.01 (1.46,2.77) | 2.01 (19.26) | 1.00 (0.50) |

SOC, system organ class; PT, preferred term; ROR, reporting odds ratio; CI, confidence interval; PRR, proportional reporting ratio; χ2, chi-squared; IC, information component; IC025, the lower limit of 95%CI of the IC.

Table S5 Signal strength of adverse events associated with micafungin at the PT level ranked by ROR in FAERS.

| SOC | PT | N | ROR (95% CI) | PRR (χ^2^) | IC (IC025) |
| --- | --- | --- | --- | --- | --- |
| Blood and lymphatic system disorders | Intravascular haemolysis | 7 | 131.73 (62.46,277.85) | 131.58 (894.85) | 7.02 (1.90) |
| Respiratory, thoracic and mediastinal disorders | Pulmonary necrosis | 3 | 93.91 (30.11,292.87) | 93.86 (272.95) | 6.54 (0.50) |
| Immune system disorders | Engraftment syndrome | 5 | 93.51 (38.74,225.72) | 93.44 (452.83) | 6.53 (1.32) |
| Renal and urinary disorders | Nephrogenic diabetes insipidus | 3 | 26.82 (8.63,83.31) | 26.81 (74.32) | 4.74 (0.40) |
| Renal and urinary disorders | Glycosuria | 3 | 23.31 (7.50,72.38) | 23.29 (63.86) | 4.54 (0.38) |
| Immune system disorders | Liver transplant rejection | 4 | 23.20 (8.69,61.91) | 23.19 (84.72) | 4.53 (0.80) |
| General disorders and administration site conditions | Multiple organ dysfunction syndrome | 92 | 22.00 (17.90,27.04) | 21.67 (1811.35) | 4.43 (3.84) |
| Blood and lymphatic system disorders | Disseminated intravascular coagulation | 27 | 19.80 (13.56,28.90) | 19.71 (478.66) | 4.30 (3.01) |
| Blood and lymphatic system disorders | Haemolysis | 14 | 19.06 (11.28,32.22) | 19.02 (238.54) | 4.25 (2.36) |
| Ear and labyrinth disorders | Ototoxicity | 3 | 17.21 (5.54,53.44) | 17.20 (45.70) | 4.10 (0.32) |
| Blood and lymphatic system disorders | Haemolytic anaemia | 13 | 15.18 (8.81,26.17) | 15.15 (171.56) | 3.92 (2.14) |
| Blood and lymphatic system disorders | Thrombotic thrombocytopenic purpura | 5 | 14.70 (6.11,35.35) | 14.69 (63.68) | 3.87 (0.98) |
| Immune system disorders | Graft versus host disease in skin | 3 | 14.49 (4.67,44.97) | 14.48 (37.59) | 3.85 (0.28) |
| Blood and lymphatic system disorders | Febrile bone marrow aplasia | 5 | 13.06 (5.43,31.41) | 13.05 (55.56) | 3.70 (0.94) |
| Gastrointestinal disorders | Pancreatitis necrotising | 3 | 12.82 (4.13,39.79) | 12.81 (32.63) | 3.68 (0.25) |
| Hepatobiliary disorders | Hepatic function abnormal | 43 | 12.63 (9.35,17.05) | 12.54 (456.39) | 3.65 (2.87) |
| Hepatobiliary disorders | Mixed liver injury | 3 | 12.42 (4.00,38.55) | 12.42 (31.45) | 3.63 (0.24) |
| Investigations | Blood bilirubin increased | 32 | 12.30 (8.69,17.41) | 12.24 (329.91) | 3.61 (2.68) |
| Blood and lymphatic system disorders | Thrombotic microangiopathy | 10 | 11.45 (6.15,21.29) | 11.43 (95.07) | 3.51 (1.68) |
| Blood and lymphatic system disorders | Haemorrhagic diathesis | 4 | 11.43 (4.29,30.48) | 11.42 (37.99) | 3.51 (0.60) |
| Respiratory, thoracic and mediastinal disorders | Acute respiratory distress syndrome | 19 | 11.34 (7.23,17.80) | 11.31 (178.39) | 3.50 (2.25) |
| General disorders and administration site conditions | Hyperpyrexia | 4 | 11.15 (4.18,29.74) | 11.15 (36.90) | 3.48 (0.59) |
| Nervous system disorders | Hypoxic-ischaemic encephalopathy | 4 | 11.01 (4.13,29.36) | 11.00 (36.33) | 3.46 (0.58) |
| Renal and urinary disorders | Renal tubular disorder | 3 | 10.80 (3.48,33.52) | 10.80 (26.64) | 3.43 (0.20) |
| Gastrointestinal disorders | Gastrointestinal necrosis | 3 | 10.73 (3.46,33.31) | 10.73 (26.44) | 3.42 (0.20) |
| Vascular disorders | Shock haemorrhagic | 8 | 10.61 (5.30,21.24) | 10.60 (69.50) | 3.40 (1.39) |
| Investigations | Aspartate aminotransferase increased | 52 | 10.28 (7.82,13.51) | 10.20 (431.20) | 3.35 (2.72) |
| Gastrointestinal disorders | Gastrointestinal perforation | 4 | 10.14 (3.80,27.03) | 10.13 (32.88) | 3.34 (0.55) |
| Investigations | Immunosuppressant drug level increased | 3 | 9.90 (3.19,30.72) | 9.90 (23.97) | 3.31 (0.17) |
| Blood and lymphatic system disorders | Agranulocytosis | 16 | 9.43 (5.77,15.41) | 9.41 (120.19) | 3.23 (1.95) |
| Hepatobiliary disorders | Hepatic failure | 27 | 9.38 (6.43,13.70) | 9.35 (201.11) | 3.22 (2.30) |
| Cardiac disorders | Left ventricular failure | 3 | 9.01 (2.90,27.96) | 9.00 (21.33) | 3.17 (0.14) |
| Investigations | Blood alkaline phosphatase increased | 21 | 8.62 (5.62,13.24) | 8.60 (140.93) | 3.10 (2.06) |
| Skin and subcutaneous tissue disorders | Haemorrhage subcutaneous | 3 | 8.58 (2.76,26.62) | 8.58 (20.06) | 3.10 (0.12) |
| Cardiac disorders | Pulseless electrical activity | 4 | 8.53 (3.20,22.75) | 8.53 (26.56) | 3.09 (0.47) |
| Blood and lymphatic system disorders | Bone marrow failure | 17 | 8.46 (5.25,13.62) | 8.44 (111.39) | 3.08 (1.90) |
| Investigations | Alanine aminotransferase increased | 48 | 8.21 (6.18,10.91) | 8.15 (301.18) | 3.03 (2.42) |
| Metabolism and nutrition disorders | Acidosis | 6 | 8.18 (3.67,18.21) | 8.17 (37.72) | 3.03 (0.92) |
| Psychiatric disorders | Dysphoria | 4 | 8.07 (3.03,21.53) | 8.07 (24.75) | 3.01 (0.45) |
| Hepatobiliary disorders | Liver injury | 16 | 7.91 (4.84,12.92) | 7.89 (96.19) | 2.98 (1.79) |
| Investigations | Blood lactate dehydrogenase increased | 12 | 7.61 (4.32,13.41) | 7.60 (68.70) | 2.92 (1.53) |
| Hepatobiliary disorders | Liver disorder | 31 | 7.49 (5.26,10.66) | 7.46 (173.32) | 2.90 (2.12) |
| Skin and subcutaneous tissue disorders | Drug eruption | 12 | 7.41 (4.21,13.06) | 7.40 (66.37) | 2.89 (1.51) |
| Respiratory, thoracic and mediastinal disorders | Respiratory failure | 50 | 7.20 (5.45,9.51) | 7.15 (264.51) | 2.84 (2.27) |
| Investigations | Blood creatine increased | 3 | 7.10 (2.29,22.03) | 7.10 (15.70) | 2.83 (0.05) |
| General disorders and administration site conditions | Hyperthermia | 5 | 6.94 (2.89,16.69) | 6.94 (25.39) | 2.79 (0.62) |
| Hepatobiliary disorders | Drug-induced liver injury | 18 | 6.86 (4.32,10.90) | 6.84 (89.77) | 2.77 (1.72) |
| Investigations | Inflammatory marker increased | 3 | 6.83 (2.20,21.18) | 6.82 (14.90) | 2.77 (0.03) |
| Renal and urinary disorders | Oliguria | 4 | 6.71 (2.52,17.89) | 6.71 (19.41) | 2.74 (0.35) |
| Cardiac disorders | Torsade de pointes | 5 | 6.71 (2.79,16.13) | 6.70 (24.25) | 2.74 (0.60) |
| Immune system disorders | Haemophagocytic lymphohistiocytosis | 6 | 6.68 (3.00,14.88) | 6.68 (28.94) | 2.74 (0.79) |
| Investigations | Gamma-glutamyltransferase increased | 14 | 6.45 (3.82,10.90) | 6.44 (64.30) | 2.69 (1.49) |
| Investigations | Breath sounds abnormal | 3 | 6.45 (2.08,20.01) | 6.45 (13.80) | 2.69 (0.00) |
| Cardiac disorders | Sinus bradycardia | 6 | 6.43 (2.89,14.32) | 6.42 (27.46) | 2.68 (0.76) |
| Hepatobiliary disorders | Hyperbilirubinaemia | 6 | 6.26 (2.81,13.95) | 6.26 (26.48) | 2.64 (0.74) |
| Vascular disorders | Shock | 13 | 6.14 (3.56,10.59) | 6.13 (55.83) | 2.62 (1.39) |
| Renal and urinary disorders | Anuria | 5 | 5.97 (2.48,14.34) | 5.96 (20.64) | 2.58 (0.53) |
| Immune system disorders | Anaphylactic shock | 14 | 5.92 (3.50,10.00) | 5.91 (57.04) | 2.56 (1.41) |
| Hepatobiliary disorders | Hepatotoxicity | 12 | 5.87 (3.33,10.34) | 5.86 (48.33) | 2.55 (1.29) |
| Metabolism and nutrition disorders | Hypokalaemia | 25 | 5.79 (3.91,8.58) | 5.77 (98.61) | 2.53 (1.72) |
| Immune system disorders | Graft versus host disease | 4 | 5.71 (2.14,15.23) | 5.71 (15.53) | 2.51 (0.26) |
| Skin and subcutaneous tissue disorders | Toxic epidermal necrolysis | 8 | 5.70 (2.85,11.40) | 5.69 (30.91) | 2.51 (0.94) |
| Respiratory, thoracic and mediastinal disorders | Lung infiltration | 5 | 5.61 (2.33,13.48) | 5.61 (18.91) | 2.49 (0.49) |
| Hepatobiliary disorders | Cholestasis | 10 | 5.58 (3.00,10.37) | 5.57 (37.48) | 2.48 (1.10) |
| Blood and lymphatic system disorders | Eosinophilia | 9 | 5.54 (2.88,10.66) | 5.54 (33.46) | 2.47 (1.02) |
| Respiratory, thoracic and mediastinal disorders | Tachypnoea | 7 | 5.52 (2.63,11.59) | 5.52 (25.89) | 2.46 (0.80) |
| Investigations | Blood urea increased | 9 | 5.41 (2.81,10.40) | 5.40 (32.26) | 2.43 (0.99) |
| Hepatobiliary disorders | Jaundice | 14 | 5.34 (3.16,9.02) | 5.33 (49.19) | 2.41 (1.30) |
| Respiratory, thoracic and mediastinal disorders | Pulmonary haemorrhage | 4 | 5.22 (1.96,13.92) | 5.22 (13.64) | 2.38 (0.21) |
| Skin and subcutaneous tissue disorders | Toxic skin eruption | 5 | 5.18 (2.15,12.45) | 5.17 (16.83) | 2.37 (0.43) |
| Investigations | Eosinophil count increased | 4 | 5.13 (1.93,13.69) | 5.13 (13.30) | 2.36 (0.20) |
| Skin and subcutaneous tissue disorders | Petechiae | 5 | 5.11 (2.12,12.27) | 5.10 (16.48) | 2.35 (0.42) |
| Respiratory, thoracic and mediastinal disorders | Pneumothorax | 8 | 5.03 (2.51,10.07) | 5.03 (25.79) | 2.33 (0.83) |
| Gastrointestinal disorders | Pancreatitis acute | 10 | 4.81 (2.59,8.95) | 4.81 (30.15) | 2.26 (0.96) |
| Gastrointestinal disorders | Intestinal perforation | 5 | 4.73 (1.97,11.38) | 4.73 (14.70) | 2.24 (0.36) |
| Investigations | Blood potassium increased | 7 | 4.53 (2.16,9.51) | 4.53 (19.23) | 2.18 (0.63) |
| Nervous system disorders | Altered state of consciousness | 9 | 4.52 (2.35,8.70) | 4.52 (24.65) | 2.18 (0.83) |
| Gastrointestinal disorders | Ileus | 5 | 4.42 (1.84,10.63) | 4.42 (13.21) | 2.14 (0.31) |
| Investigations | Blood creatinine increased | 27 | 4.30 (2.95,6.27) | 4.28 (68.00) | 2.10 (1.39) |
| Endocrine disorders | Inappropriate antidiuretic hormone secretion | 4 | 4.29 (1.61,11.43) | 4.28 (10.07) | 2.10 (0.08) |
| Renal and urinary disorders | Haematuria | 14 | 4.22 (2.50,7.13) | 4.21 (34.27) | 2.07 (1.05) |
| General disorders and administration site conditions | Mucosal inflammation | 10 | 4.09 (2.20,7.60) | 4.08 (23.26) | 2.03 (0.80) |
| Cardiac disorders | Cardiac failure | 31 | 4.07 (2.86,5.79) | 4.05 (71.39) | 2.02 (1.38) |
| Nervous system disorders | Brain injury | 4 | 4.00 (1.50,10.67) | 4.00 (8.99) | 2.00 (0.03) |
| Renal and urinary disorders | Nephropathy toxic | 4 | 3.96 (1.48,10.55) | 3.96 (8.83) | 1.98 (0.02) |
| Vascular disorders | Cyanosis | 6 | 3.95 (1.77,8.80) | 3.95 (13.21) | 1.98 (0.38) |
| Metabolism and nutrition disorders | Malnutrition | 4 | 3.86 (1.45,10.29) | 3.86 (8.46) | 1.95 (0.00) |
| Renal and urinary disorders | Renal disorder | 17 | 3.83 (2.38,6.17) | 3.83 (35.51) | 1.94 (1.04) |
| Nervous system disorders | Cerebral haemorrhage | 13 | 3.80 (2.20,6.55) | 3.79 (26.73) | 1.92 (0.89) |
| Skin and subcutaneous tissue disorders | Drug reaction with eosinophilia and systemic symptoms | 10 | 3.76 (2.02,7.00) | 3.76 (20.25) | 1.91 (0.72) |
| Hepatobiliary disorders | Hepatocellular injury | 6 | 3.70 (1.66,8.23) | 3.69 (11.78) | 1.88 (0.32) |
| Blood and lymphatic system disorders | Leukopenia | 17 | 3.62 (2.25,5.83) | 3.62 (32.18) | 1.85 (0.98) |
| Vascular disorders | Circulatory collapse | 6 | 3.62 (1.62,8.05) | 3.61 (11.33) | 1.85 (0.30) |
| Investigations | White blood cell count increased | 13 | 3.47 (2.01,5.98) | 3.46 (22.79) | 1.79 (0.79) |
| Renal and urinary disorders | Renal impairment | 27 | 3.46 (2.37,5.06) | 3.45 (47.10) | 1.79 (1.12) |
| Nervous system disorders | Cerebral infarction | 8 | 3.39 (1.69,6.78) | 3.39 (13.45) | 1.76 (0.46) |
| Skin and subcutaneous tissue disorders | Rash maculo-papular | 7 | 3.38 (1.61,7.10) | 3.38 (11.72) | 1.76 (0.36) |
| Respiratory, thoracic and mediastinal disorders | Acute respiratory failure | 6 | 3.37 (1.51,7.51) | 3.37 (10.00) | 1.75 (0.24) |
| Respiratory, thoracic and mediastinal disorders | Hypoxia | 11 | 3.35 (1.86,6.06) | 3.35 (18.14) | 1.74 (0.65) |
| Investigations | Transaminases increased | 7 | 3.32 (1.58,6.97) | 3.32 (11.34) | 1.73 (0.34) |
| Blood and lymphatic system disorders | Pancytopenia | 17 | 3.25 (2.02,5.22) | 3.24 (26.33) | 1.70 (0.84) |
| Cardiac disorders | Ventricular tachycardia | 5 | 3.17 (1.32,7.62) | 3.17 (7.43) | 1.66 (0.04) |
| Respiratory, thoracic and mediastinal disorders | Interstitial lung disease | 14 | 3.14 (1.86,5.31) | 3.14 (20.40) | 1.65 (0.71) |
| Cardiac disorders | Cardiac arrest | 25 | 3.14 (2.12,4.65) | 3.13 (36.34) | 1.65 (0.97) |
| Renal and urinary disorders | Renal failure | 41 | 3.14 (2.31,4.27) | 3.13 (59.38) | 1.64 (1.13) |
| Respiratory, thoracic and mediastinal disorders | Respiratory distress | 8 | 3.03 (1.51,6.06) | 3.03 (10.86) | 1.60 (0.34) |
| Investigations | Hepatic enzyme increased | 18 | 2.93 (1.84,4.65) | 2.92 (22.78) | 1.55 (0.74) |
| Investigations | Platelet count decreased | 28 | 2.77 (1.91,4.01) | 2.76 (31.47) | 1.46 (0.84) |
| Investigations | Blood potassium decreased | 8 | 2.77 (1.38,5.54) | 2.77 (9.02) | 1.47 (0.25) |
| Blood and lymphatic system disorders | Febrile neutropenia | 17 | 2.76 (1.72,4.45) | 2.76 (19.07) | 1.46 (0.65) |
| Investigations | International normalised ratio increased | 8 | 2.76 (1.38,5.53) | 2.76 (8.98) | 1.46 (0.24) |
| Investigations | C-reactive protein increased | 9 | 2.68 (1.39,5.16) | 2.68 (9.47) | 1.42 (0.28) |
| Investigations | Neutrophil count decreased | 10 | 2.67 (1.44,4.97) | 2.67 (10.44) | 1.42 (0.34) |
| Respiratory, thoracic and mediastinal disorders | Haemoptysis | 7 | 2.61 (1.25,5.49) | 2.61 (6.97) | 1.39 (0.10) |
| General disorders and administration site conditions | Death | 204 | 2.57 (2.24,2.96) | 2.52 (189.13) | 1.33 (1.12) |
| Gastrointestinal disorders | Gastrointestinal haemorrhage | 20 | 2.42 (1.56,3.76) | 2.42 (16.65) | 1.27 (0.55) |
| Cardiac disorders | Cardio-respiratory arrest | 10 | 2.42 (1.30,4.50) | 2.42 (8.31) | 1.27 (0.23) |
| General disorders and administration site conditions | General physical health deterioration | 23 | 2.21 (1.47,3.32) | 2.20 (15.12) | 1.14 (0.48) |
| Respiratory, thoracic and mediastinal disorders | Lung disorder | 10 | 2.18 (1.17,4.06) | 2.18 (6.41) | 1.13 (0.11) |
| Investigations | White blood cell count decreased | 22 | 2.13 (1.40,3.24) | 2.13 (13.12) | 1.09 (0.42) |
| General disorders and administration site conditions | Pyrexia | 68 | 2.08 (1.63,2.64) | 2.06 (37.49) | 1.05 (0.67) |
| Respiratory, thoracic and mediastinal disorders | Pleural effusion | 12 | 2.05 (1.16,3.60) | 2.04 (6.40) | 1.03 (0.12) |
| Blood and lymphatic system disorders | Thrombocytopenia | 21 | 2.01 (1.31,3.08) | 2.00 (10.55) | 1.00 (0.32) |

SOC, system organ class; PT, preferred term; ROR, reporting odds ratio; CI, confidence interval; PRR, proportional reporting ratio; χ^2^, chi-squared; IC, information component; IC025, the lower limit of 95%CI of the IC.

Table S6 Signal strength of adverse events associated with anidulafungin at the PT level ranked by ROR in FAERS.

| SOC | PT | N | ROR (95% CI) | PRR (χ^2^) | IC (IC025) |
| --- | --- | --- | --- | --- | --- |
| Vascular disorders | Haemodynamic instability | 4 | 32.57 (12.20,86.98) | 32.45 (121.88) | 5.02 (0.86) |
| General disorders and administration site conditions | Multiple organ dysfunction syndrome | 22 | 29.40 (19.27,44.86) | 28.81 (590.67) | 4.85 (3.10) |
| Respiratory, thoracic and mediastinal disorders | Bronchospasm | 7 | 28.39 (13.50,59.71) | 28.21 (183.66) | 4.82 (1.66) |
| Investigations | Prothrombin time prolonged | 3 | 27.24 (8.77,84.63) | 27.17 (75.58) | 4.76 (0.40) |
| Vascular disorders | Cyanosis | 7 | 25.82 (12.28,54.31) | 25.66 (165.85) | 4.68 (1.63) |
| Hepatobiliary disorders | Acute hepatic failure | 5 | 22.57 (9.37,54.34) | 22.47 (102.53) | 4.49 (1.11) |
| Respiratory, thoracic and mediastinal disorders | Tachypnoea | 4 | 17.62 (6.60,47.04) | 17.55 (62.44) | 4.13 (0.73) |
| Hepatobiliary disorders | Hepatic failure | 9 | 17.48 (9.07,33.68) | 17.33 (138.56) | 4.12 (1.80) |
| Immune system disorders | Anaphylactic shock | 7 | 16.54 (7.87,34.79) | 16.44 (101.50) | 4.04 (1.46) |
| Nervous system disorders | Status epilepticus | 3 | 15.84 (5.10,49.20) | 15.80 (41.58) | 3.98 (0.30) |
| Blood and lymphatic system disorders | Coagulopathy | 4 | 13.99 (5.24,37.34) | 13.94 (48.03) | 3.80 (0.66) |
| Investigations | Blood urea increased | 4 | 13.41 (5.02,35.80) | 13.36 (45.74) | 3.74 (0.65) |
| Hepatobiliary disorders | Cholestasis | 4 | 12.44 (4.66,33.22) | 12.40 (41.93) | 3.63 (0.62) |
| Investigations | Blood alkaline phosphatase increased | 5 | 11.44 (4.75,27.55) | 11.39 (47.40) | 3.51 (0.88) |
| Investigations | Blood bilirubin increased | 5 | 10.68 (4.44,25.72) | 10.63 (43.66) | 3.41 (0.85) |
| Respiratory, thoracic and mediastinal disorders | Respiratory failure | 13 | 10.46 (6.05,18.08) | 10.34 (109.82) | 3.37 (1.86) |
| Respiratory, thoracic and mediastinal disorders | Hypoxia | 6 | 10.23 (4.58,22.82) | 10.18 (49.66) | 3.35 (1.04) |
| Vascular disorders | Circulatory collapse | 3 | 10.08 (3.25,31.31) | 10.06 (24.47) | 3.33 (0.18) |
| Respiratory, thoracic and mediastinal disorders | Acute respiratory distress syndrome | 3 | 9.96 (3.21,30.93) | 9.93 (24.10) | 3.31 (0.17) |
| Respiratory, thoracic and mediastinal disorders | Acute respiratory failure | 3 | 9.40 (3.03,29.21) | 9.38 (22.46) | 3.23 (0.15) |
| Immune system disorders | Anaphylactic reaction | 8 | 8.90 (4.44,17.85) | 8.84 (55.70) | 3.14 (1.27) |
| Investigations | Oxygen saturation decreased | 8 | 8.72 (4.35,17.49) | 8.66 (54.28) | 3.11 (1.26) |
| Cardiac disorders | Bradycardia | 8 | 8.71 (4.34,17.47) | 8.65 (54.18) | 3.11 (1.26) |
| Hepatobiliary disorders | Hepatotoxicity | 3 | 8.17 (2.63,25.38) | 8.15 (18.82) | 3.03 (0.10) |
| Cardiac disorders | Tachycardia | 12 | 8.00 (4.53,14.13) | 7.92 (72.61) | 2.98 (1.56) |
| Investigations | Transaminases increased | 3 | 7.94 (2.56,24.66) | 7.92 (18.14) | 2.99 (0.09) |
| Respiratory, thoracic and mediastinal disorders | Respiratory disorder | 4 | 7.89 (2.96,21.07) | 7.87 (23.99) | 2.98 (0.43) |
| Investigations | Liver function test abnormal | 4 | 7.78 (2.91,20.76) | 7.75 (23.53) | 2.95 (0.43) |
| Investigations | Gamma-glutamyltransferase increased | 3 | 7.70 (2.48,23.91) | 7.68 (17.43) | 2.94 (0.08) |
| Nervous system disorders | Encephalopathy | 3 | 7.28 (2.34,22.60) | 7.26 (16.19) | 2.86 (0.05) |
| Blood and lymphatic system disorders | Thrombocytopenia | 13 | 6.97 (4.04,12.05) | 6.90 (65.70) | 2.79 (1.50) |
| Blood and lymphatic system disorders | Pancytopenia | 6 | 6.39 (2.87,14.27) | 6.36 (27.15) | 2.67 (0.75) |
| Blood and lymphatic system disorders | Leukopenia | 5 | 5.94 (2.47,14.31) | 5.92 (20.46) | 2.57 (0.52) |
| Musculoskeletal and connective tissue disorders | Rhabdomyolysis | 4 | 5.70 (2.14,15.22) | 5.68 (15.45) | 2.51 (0.26) |
| Investigations | Hepatic enzyme increased | 6 | 5.45 (2.44,12.16) | 5.42 (21.66) | 2.44 (0.64) |
| Respiratory, thoracic and mediastinal disorders | Pulmonary oedema | 4 | 5.18 (1.94,13.84) | 5.17 (13.45) | 2.37 (0.20) |
| Metabolism and nutrition disorders | Hypokalaemia | 4 | 5.15 (1.93,13.76) | 5.14 (13.34) | 2.36 (0.20) |
| Investigations | Alanine aminotransferase increased | 5 | 4.74 (1.97,11.42) | 4.72 (14.69) | 2.24 (0.36) |
| Gastrointestinal disorders | Pancreatitis | 4 | 4.45 (1.67,11.87) | 4.43 (10.64) | 2.15 (0.10) |
| Investigations | Blood creatinine increased | 5 | 4.43 (1.84,10.67) | 4.42 (13.22) | 2.14 (0.31) |
| Investigations | Aspartate aminotransferase increased | 4 | 4.38 (1.64,11.68) | 4.36 (10.38) | 2.13 (0.09) |
| Cardiac disorders | Cardiac arrest | 6 | 4.20 (1.88,9.38) | 4.19 (14.57) | 2.07 (0.43) |
| Cardiac disorders | Cardiac failure | 5 | 3.65 (1.52,8.79) | 3.64 (9.59) | 1.86 (0.16) |
| Nervous system disorders | Seizure | 10 | 3.40 (1.82,6.34) | 3.38 (16.80) | 1.76 (0.60) |
| Vascular disorders | Hypotension | 11 | 3.24 (1.79,5.87) | 3.22 (16.88) | 1.69 (0.60) |
| Skin and subcutaneous tissue disorders | Erythema | 11 | 3.08 (1.70,5.58) | 3.06 (15.27) | 1.61 (0.54) |
| Renal and urinary disorders | Acute kidney injury | 10 | 3.00 (1.61,5.60) | 2.99 (13.25) | 1.58 (0.46) |
| Respiratory, thoracic and mediastinal disorders | Dyspnoea | 28 | 2.96 (2.03,4.30) | 2.90 (35.29) | 1.54 (0.90) |
| General disorders and administration site conditions | Death | 39 | 2.74 (1.99,3.78) | 2.68 (41.62) | 1.42 (0.90) |
| Vascular disorders | Hypertension | 8 | 2.23 (1.11,4.47) | 2.22 (5.38) | 1.15 (0.00) |

SOC, system organ class; PT, preferred term; ROR, reporting odds ratio; CI, confidence interval; PRR, proportional reporting ratio; χ2, chi-squared; IC, information component; IC025, the lower limit of 95%CI of the IC.

Table S7 Multivariable logistic regression analyses of hospitalization-associated reporting adjusted for age and sex.

| **PT** | **aOR** | **95% CI** | ***P* value** |
| --- | --- | --- | --- |
| **Caspofungin** | | | |
| Multiple organ dysfunction syndrome | 1.42 | 0.94-2.14 | 0.097 |
| Blood alkaline phosphatase increased | 2.09 | 1.12-3.89 | 0.020 |
| Drug reaction with eosinophilia and systemic symptoms | 6.77 | 3.22-14.24 | <0.001 |
| Alanine aminotransferase increased | 1.89 | 1.00-3.57 | 0.050 |
| Aspartate aminotransferase increased | 2.36 | 1.22-4.55 | 0.011 |
| Acute kidney injury | 2.14 | 1.09-4.19 | 0.027 |
| C-reactive protein increased | 2.25 | 1.10-4.60 | 0.026 |
| Gamma-glutamyltransferase increased | 4.36 | 1.90-10.03 | 0.001 |
| Hepatic function abnormal | 2.27 | 1.08-4.75 | 0.030 |
| **Micafungin** | | | |
| Death | 0.32 | 0.16-0.65 | 0.001 |
| Pyrexia | 2.13 | 1.20-3.81 | 0.010 |
| Respiratory failure | 1.61 | 0.84-3.06 | 0.149 |
| Renal failure | 2.07 | 1.04-4.12 | 0.039 |
| Hepatic failure | 2.60 | 1.10-6.15 | 0.030 |
| General physical health deterioration | 2.09 | 0.87-5.05 | 0.100 |
| **Anidulafungin** | | | |
| Death | 0.39 | 0.09-1.77 | 0.221 |
| Hepatic failure | 20.35 | 2.21-187.69 | 0.008 |
| Anaphylactic shock | 2.81 | 0.37-21.09 | 0.315 |

PT, preferred term; aOR, adjusted odds ratio; CI, confidence interval.

Table S8 Top 30 positive signals for echinocandins-related adverse events from healthcare professional in FAERS.

| No. | PT | N | ROR (95%CI lower) | PT | N | ROR (95%CI lower) | PT | N | ROR (95%CI lower) |
| --- | --- | --- | --- | --- | --- | --- | --- | --- | --- |
| 1 | Respiratory failure | 45 | 4.79(3.57,6.42) | Aspartate aminotransferase increased | 40 | 7.20(5.27,9.83) | Dyspnoea | 24 | 3.02(2.01,4.53) |
| 2 | Drug reaction with eosinophilia and systemic symptoms | 45 | 10.55(7.87,14.16) | Respiratory failure | 38 | 5.17(3.76,7.12) | Multiple organ dysfunction syndrome | 20 | 19.59(12.57,30.52) |
| 3 | Cholestasis | 43 | 15.27(11.30,20.62) | Hepatic function abnormal | 36 | 9.39(6.76,13.04) | Tachycardia | 12 | 6.66(3.77,11.77) |
| 4 | Blood alkaline phosphatase increased | 37 | 11.37(8.23,15.72) | Alanine aminotransferase increased | 36 | 5.59(4.02,7.76) | Erythema | 10 | 3.80(2.03,7.08) |
| 5 | Renal failure | 36 | 3.15(2.27,4.37) | Renal failure | 32 | 3.58(2.53,5.07) | Respiratory failure | 10 | 6.14(3.29,11.46) |
| 6 | Alanine aminotransferase increased | 35 | 4.24(3.04,5.91) | Liver disorder | 26 | 7.90(5.37,11.62) | Seizure | 10 | 3.80(2.04,7.09) |
| 7 | Aspartate aminotransferase increased | 31 | 4.35(3.05,6.19) | Blood bilirubin increased | 25 | 9.11(6.15,13.50) | Thrombocytopenia | 10 | 3.67(1.96,6.84) |
| 8 | Pancytopenia | 28 | 3.46(2.38,5.01) | Renal impairment | 23 | 3.23(2.15,4.87) | Hypotension | 9 | 2.35(1.22,4.53) |
| 9 | Eosinophilia | 27 | 10.86(7.44,15.86) | Hepatic failure | 23 | 7.85(5.21,11.83) | Anaphylactic reaction | 8 | 7.00(3.49,14.03) |
| 10 | Gamma-glutamyltransferase increased | 24 | 7.83(5.24,11.70) | Disseminated intravascular coagulation | 22 | 13.57(8.92,20.64) | Bradycardia | 8 | 6.28(3.13,12.59) |
| 11 | Graft versus host disease | 24 | 24.28(16.24,36.29) | Cardiac arrest | 21 | 2.59(1.69,3.98) | Hepatic failure | 8 | 12.33(6.15,24.74) |
| 12 | Hypokalaemia | 24 | 3.83(2.57,5.73) | Blood alkaline phosphatase increased | 21 | 8.22(5.36,12.63) | Oxygen saturation decreased | 8 | 8.10(4.04,16.25) |
| 13 | Agranulocytosis | 23 | 8.51(5.65,12.82) | General physical health deterioration | 20 | 2.19(1.41,3.39) | Bronchospasm | 7 | 23.26(11.05,48.96) |
| 14 | Blood lactate dehydrogenase increased | 22 | 10.51(6.91,15.98) | Acute respiratory distress syndrome | 19 | 9.59(6.11,15.05) | Cardiac arrest | 6 | 3.34(1.50,7.46) |
| 15 | C-reactive protein increased | 22 | 4.64(3.06,7.06) | Cardiac failure | 16 | 2.26(1.38,3.70) | Cyanosis | 6 | 18.30(8.20,40.86) |
| 16 | Hepatic failure | 22 | 5.86(3.86,8.92) | Gamma-glutamyltransferase increased | 14 | 5.83(3.45,9.85) | Hepatic enzyme increased | 6 | 5.21(2.33,11.63) |
| 17 | Rash maculo-papular | 22 | 7.09(4.66,10.78) | Shock | 13 | 5.59(3.24,9.64) | Acute hepatic failure | 5 | 14.82(6.15,35.69) |
| 18 | Blood bilirubin increased | 21 | 5.97(3.89,9.17) | Pancytopenia | 13 | 2.05(1.19,3.53) | Blood bilirubin increased | 5 | 8.20(3.40,19.74) |
| 19 | Hepatic function abnormal | 21 | 4.26(2.78,6.54) | Renal disorder | 12 | 5.12(2.90,9.02) | Blood creatinine increased | 5 | 3.71(1.54,8.94) |
| 20 | Acute respiratory distress syndrome | 20 | 7.89(5.08,12.24) | Leukopenia | 12 | 2.16(1.23,3.82) | Cardiac failure | 5 | 3.19(1.32,7.68) |
| 21 | Transaminases increased | 20 | 6.33(4.08,9.82) | Drug-induced liver injury | 12 | 3.71(2.10,6.54) | Hypoxia | 5 | 6.36(2.64,15.31) |
| 22 | Drug-induced liver injury | 20 | 4.84(3.12,7.51) | Cerebral haemorrhage | 12 | 3.80(2.15,6.69) | Pancytopenia | 5 | 3.55(1.48,8.56) |
| 23 | Hepatocellular injury | 18 | 7.39(4.65,11.75) | Jaundice | 11 | 4.13(2.29,7.47) | Blood alkaline phosphatase increased | 5 | 8.82(3.66,21.25) |
| 24 | Liver disorder | 18 | 4.26(2.68,6.77) | Hypoxia | 11 | 3.10(1.71,5.60) | Anaphylactic shock | 4 | 7.68(2.87,20.50) |
| 25 | Disseminated intravascular coagulation | 17 | 8.18(5.08,13.18) | Hepatotoxicity | 11 | 4.48(2.48,8.11) | Blood urea increased | 4 | 10.72(4.01,28.64) |
| 26 | White blood cell count increased | 17 | 4.16(2.58,6.70) | Haematuria | 11 | 3.56(1.97,6.44) | Cholestasis | 4 | 8.13(3.04,21.70) |
| 27 | Toxic skin eruption | 17 | 11.53(7.16,18.58) | Blood lactate dehydrogenase increased | 11 | 6.70(3.71,12.11) | Coagulopathy | 4 | 12.25(4.59,32.73) |
| 28 | Hepatotoxicity | 16 | 5.11(3.13,8.35) | Anaphylactic shock | 11 | 4.68(2.59,8.46) | Liver function test abnormal | 4 | 6.44(2.41,17.19) |
| 29 | Liver injury | 16 | 6.30(3.85,10.29) | White blood cell count increased | 10 | 3.12(1.68,5.81) | Pancreatitis | 4 | 4.56(1.71,12.17) |
| 30 | Encephalopathy | 15 | 4.30(2.59,7.14) | Mucosal inflammation | 10 | 3.49(1.88,6.49) | Pulmonary oedema | 4 | 5.19(1.94,13.87) |

PT, preferred term; ROR, reporting odds ratio; CI, confidence interval.

Table S9 Signal strength of adverse events associated with caspofungin at the PT level ranked by ROR in Vigiaccess.

| SOC | PT | N | ROR (95% CI) | PRR (χ^2^) | IC (IC025) |
| --- | --- | --- | --- | --- | --- |
| Skin and subcutaneous tissue disorders | Cutaneous calcification | 15 | 1042.63(617.83,1759.53) | 1040.58(14596.9) | 9.93(3.23) |
| Investigations | Blood alkaline phosphatase abnormal | 3 | 56.97(18.33,177.04) | 56.94(164.28) | 5.83(0.48) |
| Hepatobiliary disorders | Hepatotoxicity | 60 | 33.91(26.29,43.72) | 33.65(1896.95) | 5.07(4.08) |
| General disorders and administration site conditions | Multiple organ dysfunction syndrome | 86 | 32.37(26.17,40.05) | 32.02(2579.69) | 5.00(4.25) |
| Hepatobiliary disorders | Cholestasis | 44 | 28.19(20.95,37.92) | 28.03(1145.10) | 4.81(3.70) |
| Hepatobiliary disorders | Hyperbilirubinaemia | 48 | 26.33(19.82,34.98) | 26.17(1160.46) | 4.71(3.70) |
| Hepatobiliary disorders | Mixed liver injury | 6 | 25.60(11.49,57.05) | 25.58(141.51) | 4.67(1.41) |
| Renal and urinary disorders | Nephrogenic diabetes insipidus | 3 | 25.38(8.18,78.80) | 25.37(70.13) | 4.66(0.39) |
| Renal and urinary disorders | Renal tubular disorder | 6 | 23.76(10.67,52.94) | 23.75(130.53) | 4.57(1.39) |
| Investigations | Blood alkaline phosphatase increased | 65 | 22.53(17.65,28.77) | 22.35(1324.09) | 4.48(3.72) |
| Investigations | Blood bilirubin increased | 63 | 22.29(17.39,28.56) | 22.11(1268.54) | 4.46(3.69) |
| Immune system disorders | Acute graft versus host disease | 5 | 20.82(8.66,50.07) | 20.81(94.17) | 4.38(1.09) |
| Immune system disorders | Graft versus host disease | 11 | 20.47(11.33,37.00) | 20.45(203.19) | 4.35(2.13) |
| Endocrine disorders | Diabetes insipidus | 4 | 20.01(7.50,53.36) | 20.00(72.11) | 4.32(0.77) |
| Respiratory, thoracic and mediastinal disorders | Hypercapnia | 4 | 18.38(6.89,49.02) | 18.37(65.63) | 4.20(0.75) |
| Gastrointestinal disorders | Enterocolitis | 8 | 17.25(8.62,34.52) | 17.23(122.19) | 4.11(1.66) |
| Metabolism and nutrition disorders | Hypokalaemia | 119 | 17.00(14.19,20.38) | 16.76(1762.69) | 4.07(3.62) |
| Hepatobiliary disorders | Cholestatic liver injury | 3 | 16.86(5.43,52.33) | 16.86(44.70) | 4.07(0.32) |
| Skin and subcutaneous tissue disorders | Toxic skin eruption | 21 | 16.85(10.98,25.86) | 16.80(311.86) | 4.07(2.67) |
| Nervous system disorders | Cerebellar syndrome | 3 | 16.58(5.34,51.44) | 16.57(43.84) | 4.05(0.32) |
| Metabolism and nutrition disorders | Hyperphosphataemia | 3 | 16.49(5.31,51.16) | 16.48(43.58) | 4.04(0.31) |
| Immune system disorders | Haemophagocytic lymphohistiocytosis | 10 | 16.34(8.78,30.39) | 16.32(143.66) | 4.03(1.90) |
| Metabolism and nutrition disorders | Hypercalcaemia | 18 | 16.17(10.18,25.68) | 16.13(255.22) | 4.01(2.50) |
| Investigations | Blood lactate dehydrogenase increased | 21 | 15.79(10.29,24.24) | 15.75(289.90) | 3.98(2.62) |
| Skin and subcutaneous tissue disorders | Toxic epidermal necrolysis | 34 | 15.25(10.89,21.37) | 15.19(450.43) | 3.92(2.94) |
| Metabolism and nutrition disorders | Hypomagnesaemia | 16 | 15.18(9.29,24.80) | 15.15(211.29) | 3.92(2.35) |
| Hepatobiliary disorders | Hypertransaminasaemia | 14 | 14.68(8.69,24.80) | 14.65(177.93) | 3.87(2.19) |
| Respiratory, thoracic and mediastinal disorders | Acute respiratory distress syndrome | 20 | 14.32(9.23,22.21) | 14.29(246.93) | 3.84(2.50) |
| Blood and lymphatic system disorders | Eosinophilia | 39 | 14.08(10.28,19.29) | 14.02(471.14) | 3.81(2.94) |
| Skin and subcutaneous tissue disorders | Drug reaction with eosinophilia and systemic symptoms | 39 | 13.97(10.20,19.14) | 13.91(466.88) | 3.80(2.94) |
| Respiratory, thoracic and mediastinal disorders | Pulmonary alveolar haemorrhage | 5 | 13.78(5.73,33.12) | 13.77(59.15) | 3.78(0.96) |
| Hepatobiliary disorders | Hepatic cytolysis | 28 | 13.41(9.25,19.44) | 13.37(320.14) | 3.74(2.69) |
| Respiratory, thoracic and mediastinal disorders | Pulmonary haemorrhage | 9 | 12.85(6.68,24.71) | 12.83(98.14) | 3.68(1.64) |
| Skin and subcutaneous tissue disorders | Dermatitis exfoliative generalised | 10 | 12.54(6.75,23.33) | 12.53(106.02) | 3.65(1.74) |
| Investigations | Transaminases increased | 44 | 12.48(9.27,16.78) | 12.41(461.38) | 3.63(2.87) |
| Investigations | Gamma-glutamyltransferase increased | 35 | 12.44(8.92,17.34) | 12.39(366.28) | 3.63(2.75) |
| General disorders and administration site conditions | Organ failure | 3 | 12.39(3.99,38.44) | 12.39(31.38) | 3.63(0.24) |
| Hepatobiliary disorders | Liver injury | 29 | 11.57(8.03,16.66) | 11.53(278.78) | 3.53(2.56) |
| Respiratory, thoracic and mediastinal disorders | Respiratory acidosis | 3 | 11.46(3.69,35.56) | 11.46(28.61) | 3.52(0.22) |
| Respiratory, thoracic and mediastinal disorders | Acute pulmonary oedema | 4 | 11.41(4.28,30.43) | 11.41(37.96) | 3.51(0.60) |
| Hepatobiliary disorders | Venoocclusive liver disease | 5 | 11.37(4.73,27.34) | 11.36(47.23) | 3.51(0.88) |
| Hepatobiliary disorders | Hepatitis fulminant | 3 | 11.03(3.56,34.22) | 11.03(27.33) | 3.46(0.21) |
| General disorders and administration site conditions | Systemic inflammatory response syndrome | 3 | 10.89(3.51,33.79) | 10.89(26.92) | 3.44(0.20) |
| Investigations | Aspartate aminotransferase increased | 75 | 10.88(8.67,13.66) | 10.78(665.85) | 3.43(2.92) |
| Investigations | Hepatic enzyme increased | 97 | 10.84(8.87,13.24) | 10.71(854.35) | 3.42(2.99) |
| Hepatobiliary disorders | Hepatic failure | 26 | 10.82(7.36,15.90) | 10.78(230.67) | 3.43(2.43) |
| Hepatobiliary disorders | Hepatitis cholestatic | 15 | 10.65(6.42,17.68) | 10.63(130.80) | 3.41(2.01) |
| Hepatobiliary disorders | Hepatic necrosis | 4 | 10.64(3.99,28.36) | 10.63(34.88) | 3.41(0.57) |
| Investigations | Liver function test abnormal | 36 | 10.57(7.62,14.66) | 10.52(310.12) | 3.39(2.59) |
| Renal and urinary disorders | Cystitis haemorrhagic | 4 | 10.37(3.89,27.64) | 10.36(33.81) | 3.37(0.56) |
| Hepatobiliary disorders | Hepatic function abnormal | 106 | 10.28(8.49,12.45) | 10.15(875.23) | 3.34(2.94) |
| Investigations | Neutrophil count increased | 9 | 9.67(5.03,18.60) | 9.66(69.87) | 3.27(1.46) |
| Investigations | Alanine aminotransferase increased | 80 | 9.54(7.65,11.89) | 9.45(604.58) | 3.24(2.77) |
| Investigations | Coagulation time prolonged | 5 | 9.38(3.90,22.55) | 9.38(37.39) | 3.23(0.79) |
| Skin and subcutaneous tissue disorders | Acute generalised exanthematous pustulosis | 8 | 9.37(4.68,18.74) | 9.36(59.70) | 3.23(1.32) |
| Skin and subcutaneous tissue disorders | Rash morbilliform | 12 | 9.03(5.12,15.90) | 9.01(85.46) | 3.17(1.68) |
| Hepatobiliary disorders | Acute hepatic failure | 8 | 8.91(4.45,17.83) | 8.90(56.10) | 3.15(1.28) |
| Blood and lymphatic system disorders | Agranulocytosis | 37 | 8.67(6.27,11.97) | 8.63(249.57) | 3.11(2.38) |
| Hepatobiliary disorders | Hepatocellular injury | 13 | 8.54(4.95,14.71) | 8.53(86.33) | 3.09(1.70) |
| Metabolism and nutrition disorders | Hypernatraemia | 4 | 8.37(3.14,22.32) | 8.37(25.94) | 3.06(0.47) |
| Hepatobiliary disorders | Drug-induced liver injury | 17 | 8.23(5.12,13.25) | 8.22(107.74) | 3.04(1.87) |
| Blood and lymphatic system disorders | Disseminated intravascular coagulation | 8 | 8.14(4.07,16.29) | 8.13(50.02) | 3.02(1.22) |
| Metabolism and nutrition disorders | Hypoalbuminaemia | 4 | 7.95(2.98,21.19) | 7.95(24.28) | 2.99(0.44) |
| Metabolism and nutrition disorders | Electrolyte imbalance | 8 | 7.78(3.89,15.56) | 7.77(47.16) | 2.96(1.19) |
| Renal and urinary disorders | Nephropathy toxic | 8 | 7.73(3.87,15.48) | 7.73(46.84) | 2.95(1.18) |
| Respiratory, thoracic and mediastinal disorders | Respiratory failure | 40 | 7.72(5.66,10.53) | 7.68(232.53) | 2.94(2.27) |
| Immune system disorders | Transplant rejection | 5 | 7.70(3.20,18.51) | 7.70(29.12) | 2.94(0.68) |
| Vascular disorders | Haemodynamic instability | 3 | 7.62(2.46,23.63) | 7.61(17.23) | 2.93(0.08) |
| Nervous system disorders | Hydrocephalus | 3 | 7.55(2.43,23.43) | 7.55(17.04) | 2.92(0.07) |
| Immune system disorders | Hypogammaglobulinaemia | 3 | 7.54(2.43,23.40) | 7.54(17.01) | 2.91(0.07) |
| Gastrointestinal disorders | Ileus paralytic | 3 | 7.26(2.34,22.52) | 7.26(16.18) | 2.86(0.06) |
| Hepatobiliary disorders | Jaundice cholestatic | 3 | 7.04(2.27,21.83) | 7.04(15.53) | 2.81(0.04) |
| Respiratory, thoracic and mediastinal disorders | Pneumothorax | 8 | 6.85(3.42,13.70) | 6.84(39.90) | 2.77(1.09) |
| Blood and lymphatic system disorders | Aplastic anaemia | 4 | 6.81(2.55,18.14) | 6.80(19.80) | 2.77(0.36) |
| Respiratory, thoracic and mediastinal disorders | Respiratory distress | 20 | 6.57(4.23,10.18) | 6.55(94.07) | 2.71(1.74) |
| Investigations | Aspartate aminotransferase | 3 | 6.42(2.07,19.92) | 6.42(13.72) | 2.68(0.00) |
| Metabolism and nutrition disorders | Acidosis | 5 | 6.18(2.57,14.86) | 6.18(21.69) | 2.63(0.55) |
| Blood and lymphatic system disorders | Pancytopenia | 38 | 6.16(4.48,8.48) | 6.14(163.53) | 2.62(1.98) |
| Nervous system disorders | Encephalopathy | 16 | 6.16(3.77,10.07) | 6.15(69.04) | 2.62(1.54) |
| Hepatobiliary disorders | Liver disorder | 18 | 6.09(3.83,9.67) | 6.08(76.34) | 2.60(1.60) |
| Blood and lymphatic system disorders | Thrombocytopenia | 132 | 6.08(5.12,7.22) | 5.99(550.43) | 2.58(2.28) |
| Skin and subcutaneous tissue disorders | Rash maculo-papular | 120 | 5.97(4.98,7.15) | 5.89(488.44) | 2.56(2.24) |
| Metabolism and nutrition disorders | Cachexia | 4 | 5.96(2.23,15.88) | 5.95(16.48) | 2.57(0.29) |
| Hepatobiliary disorders | Hepatitis acute | 4 | 5.80(2.18,15.46) | 5.80(15.87) | 2.53(0.27) |
| Investigations | Liver function test increased | 12 | 5.78(3.28,10.19) | 5.77(47.37) | 2.53(1.28) |
| Blood and lymphatic system disorders | Bone marrow failure | 6 | 5.77(2.59,12.85) | 5.77(23.63) | 2.53(0.69) |
| Investigations | C-reactive protein increased | 16 | 5.74(3.52,9.38) | 5.73(62.52) | 2.52(1.46) |
| Renal and urinary disorders | Renal impairment | 47 | 5.69(4.27,7.58) | 5.66(180.44) | 2.50(1.95) |
| Hepatobiliary disorders | Hepatitis | 30 | 5.59(3.90,8.00) | 5.57(112.48) | 2.48(1.76) |
| Cardiac disorders | Atrioventricular block | 5 | 5.37(2.24,12.92) | 5.37(17.79) | 2.43(0.46) |
| Respiratory, thoracic and mediastinal disorders | Pulmonary mass | 5 | 5.34(2.22,12.83) | 5.34(17.61) | 2.42(0.45) |
| Nervous system disorders | Central nervous system lesion | 5 | 5.32(2.21,12.78) | 5.31(17.51) | 2.41(0.45) |
| Skin and subcutaneous tissue disorders | Stevens-Johnson syndrome | 25 | 5.18(3.50,7.68) | 5.17(84.09) | 2.37(1.59) |
| Investigations | Blood potassium decreased | 12 | 5.12(2.90,9.01) | 5.11(39.67) | 2.35(1.16) |
| Nervous system disorders | Brain oedema | 5 | 5.07(2.11,12.20) | 5.07(16.34) | 2.34(0.42) |
| Cardiac disorders | Sinus bradycardia | 5 | 5.04(2.09,12.10) | 5.03(16.15) | 2.33(0.41) |
| Renal and urinary disorders | Renal tubular necrosis | 4 | 4.88(1.83,13.01) | 4.88(12.33) | 2.29(0.17) |
| Blood and lymphatic system disorders | Coagulopathy | 10 | 4.68(2.51,8.70) | 4.67(28.85) | 2.22(0.94) |
| Cardiac disorders | Ventricular fibrillation | 5 | 4.58(1.91,11.01) | 4.58(13.97) | 2.19(0.34) |
| Hepatobiliary disorders | Jaundice | 19 | 4.26(2.72,6.68) | 4.25(47.26) | 2.09(1.22) |
| Blood and lymphatic system disorders | Febrile neutropenia | 32 | 4.19(2.96,5.93) | 4.18(77.36) | 2.06(1.43) |
| Skin and subcutaneous tissue disorders | Drug eruption | 14 | 4.17(2.47,7.05) | 4.17(33.73) | 2.06(1.04) |
| Blood and lymphatic system disorders | Lymphopenia | 7 | 3.94(1.88,8.28) | 3.94(15.36) | 1.98(0.51) |
| Investigations | Blood creatinine increased | 25 | 3.81(2.58,5.65) | 3.81(51.74) | 1.93(1.21) |
| Nervous system disorders | Cerebral infarction | 8 | 3.78(1.89,7.56) | 3.78(16.32) | 1.92(0.57) |
| Renal and urinary disorders | Renal failure | 46 | 3.55(2.66,4.75) | 3.54(83.81) | 1.82(1.32) |
| Vascular disorders | Phlebitis | 8 | 3.54(1.77,7.08) | 3.54(14.55) | 1.82(0.50) |
| Investigations | Electrocardiogram QT prolonged | 11 | 3.44(1.91,6.22) | 3.44(19.04) | 1.78(0.68) |
| Metabolism and nutrition disorders | Metabolic acidosis | 6 | 3.40(1.53,7.57) | 3.40(10.15) | 1.76(0.25) |
| Respiratory, thoracic and mediastinal disorders | Respiratory disorder | 9 | 3.28(1.70,6.30) | 3.27(14.21) | 1.71(0.50) |
| Vascular disorders | Shock | 7 | 3.25(1.55,6.82) | 3.25(10.90) | 1.70(0.32) |
| Renal and urinary disorders | Acute kidney injury | 60 | 3.07(2.38,3.96) | 3.06(83.17) | 1.61(1.19) |
| Respiratory, thoracic and mediastinal disorders | Bronchospasm | 12 | 2.92(1.66,5.15) | 2.92(15.16) | 1.55(0.55) |
| Gastrointestinal disorders | Pancreatitis | 14 | 2.91(1.72,4.92) | 2.91(17.53) | 1.54(0.62) |
| Gastrointestinal disorders | Pancreatitis acute | 6 | 2.89(1.30,6.42) | 2.88(7.38) | 1.53(0.09) |
| Investigations | White blood cell count increased | 10 | 2.88(1.55,5.36) | 2.88(12.27) | 1.53(0.43) |
| Psychiatric disorders | Delirium | 10 | 2.86(1.54,5.33) | 2.86(12.12) | 1.52(0.42) |
| Respiratory, thoracic and mediastinal disorders | Pleural effusion | 13 | 2.86(1.66,4.92) | 2.85(15.67) | 1.51(0.56) |
| Blood and lymphatic system disorders | Neutropenia | 56 | 2.79(2.15,3.63) | 2.78(63.92) | 1.47(1.05) |
| Cardiac disorders | Cardio-respiratory arrest | 11 | 2.76(1.53,4.99) | 2.76(12.36) | 1.46(0.43) |
| Skin and subcutaneous tissue disorders | Purpura | 8 | 2.74(1.37,5.48) | 2.74(8.84) | 1.45(0.24) |
| Respiratory, thoracic and mediastinal disorders | Interstitial lung disease | 13 | 2.60(1.51,4.49) | 2.60(12.81) | 1.38(0.45) |
| General disorders and administration site conditions | Mucosal inflammation | 8 | 2.58(1.29,5.16) | 2.58(7.71) | 1.36(0.17) |
| Investigations | Oxygen saturation decreased | 13 | 2.52(1.46,4.35) | 2.52(11.92) | 1.33(0.41) |
| Cardiac disorders | Cardiac failure | 13 | 2.17(1.26,3.75) | 2.17(8.23) | 1.12(0.23) |
| Blood and lymphatic system disorders | Leukopenia | 26 | 2.08(1.42,3.06) | 2.08(14.56) | 1.05(0.44) |

SOC, system organ class; PT, preferred term; ROR, reporting odds ratio; CI, confidence interval; PRR, proportional reporting ratio; χ2, chi-squared; IC, information component; IC025, the lower limit of 95%CI of the IC.

Table S10 Signal strength of adverse events associated with micafungin at the PT level ranked by ROR in Vigiaccess.

| SOC | PT | N | ROR (95% CI) | PRR (χ^2^) | IC (IC025) |
| --- | --- | --- | --- | --- | --- |
| Blood and lymphatic system disorders | Intravascular haemolysis | 8 | 204.11(101.58,410.13) | 203.89(1594.40) | 7.65(2.14) |
| Musculoskeletal and connective tissue disorders | Soft tissue necrosis | 3 | 60.29(19.40,187.38) | 60.26(174.17) | 5.91(0.48) |
| Immune system disorders | Lung transplant rejection | 3 | 56.72(18.25,176.27) | 56.70(163.55) | 5.82(0.48) |
| Hepatobiliary disorders | Cholestatic liver injury | 7 | 39.88(18.99,83.77) | 39.85(264.44) | 5.31(1.74) |
| General disorders and administration site conditions | Infusion site warmth | 3 | 32.57(10.49,101.12) | 32.56(91.57) | 5.02(0.43) |
| Blood and lymphatic system disorders | Haemolysis | 20 | 28.22(18.19,43.78) | 28.14(522.72) | 4.81(2.99) |
| Immune system disorders | Graft versus host disease in gastrointestinal tract | 4 | 28.20(10.57,75.22) | 28.18(104.69) | 4.81(0.84) |
| Metabolism and nutrition disorders | Hypoproteinaemia | 4 | 27.37(10.26,73.01) | 27.36(101.40) | 4.77(0.83) |
| General disorders and administration site conditions | Multiple organ dysfunction syndrome | 72 | 27.36(21.69,34.51) | 27.10(1807.72) | 4.76(3.98) |
| Immune system disorders | Graft versus host disease in skin | 4 | 27.30(10.23,72.82) | 27.29(101.12) | 4.77(0.83) |
| Gastrointestinal disorders | Neutropenic colitis | 4 | 27.19(10.19,72.54) | 27.18(100.69) | 4.76(0.83) |
| Immune system disorders | Acute graft versus host disease in skin | 4 | 24.95(9.35,66.55) | 24.94(91.77) | 4.64(0.81) |
| Immune system disorders | Graft versus host disease | 13 | 24.49(14.21,42.21) | 24.45(291.92) | 4.61(2.42) |
| Renal and urinary disorders | Glycosuria | 4 | 22.38(8.39,59.69) | 22.37(81.55) | 4.48(0.79) |
| Immune system disorders | Transplant rejection | 14 | 21.86(12.93,36.93) | 21.82(277.71) | 4.45(2.44) |
| Hepatobiliary disorders | Mixed liver injury | 5 | 21.57(8.97,51.88) | 21.56(97.90) | 4.43(1.10) |
| Investigations | Blood bilirubin increased | 58 | 20.74(16.02,26.86) | 20.59(1079.98) | 4.36(3.57) |
| Blood and lymphatic system disorders | Haemolytic anaemia | 24 | 19.60(13.13,29.27) | 19.54(421.84) | 4.29(2.91) |
| General disorders and administration site conditions | Mucosal inflammation | 59 | 19.38(15.00,25.04) | 19.23(1019.01) | 4.26(3.51) |
| Investigations | Immunosuppressant drug level increased | 3 | 17.84(5.75,55.37) | 17.84(47.62) | 4.16(0.33) |
| Investigations | Bilirubin conjugated increased | 3 | 17.00(5.48,52.77) | 17.00(45.12) | 4.09(0.32) |
| Blood and lymphatic system disorders | Disseminated intravascular coagulation | 16 | 16.49(10.10,26.94) | 16.46(232.14) | 4.04(2.41) |
| Investigations | Sputum abnormal | 3 | 16.39(5.28,50.85) | 16.38(43.28) | 4.03(0.31) |
| Hepatobiliary disorders | Hyperbilirubinaemia | 28 | 15.49(10.68,22.45) | 15.43(377.66) | 3.95(2.83) |
| Hepatobiliary disorders | Liver disorder | 45 | 15.46(11.53,20.73) | 15.38(604.45) | 3.94(3.12) |
| Immune system disorders | Haemophagocytic lymphohistiocytosis | 9 | 14.87(7.73,28.60) | 14.85(116.20) | 3.89(1.72) |
| Blood and lymphatic system disorders | Thrombotic thrombocytopenic purpura | 5 | 14.67(6.10,35.28) | 14.66(63.60) | 3.87(0.98) |
| Blood and lymphatic system disorders | Erythropenia | 3 | 14.20(4.58,44.05) | 14.19(36.75) | 3.83(0.28) |
| Investigations | Aspartate aminotransferase increased | 96 | 14.13(11.55,17.29) | 13.96(1155.52) | 3.80(3.33) |
| Hepatobiliary disorders | Drug-induced liver injury | 28 | 13.74(9.48,19.92) | 13.70(329.33) | 3.77(2.71) |
| Blood and lymphatic system disorders | Thrombotic microangiopathy | 8 | 13.66(6.82,27.33) | 13.64(93.66) | 3.77(1.54) |
| Investigations | Alanine aminotransferase increased | 108 | 13.08(10.81,15.81) | 12.90(1186.37) | 3.69(3.26) |
| Ear and labyrinth disorders | Ototoxicity | 3 | 13.07(4.21,40.57) | 13.07(33.41) | 3.71(0.26) |
| Immune system disorders | Acute graft versus host disease | 3 | 12.63(4.07,39.18) | 12.62(32.08) | 3.66(0.25) |
| Respiratory, thoracic and mediastinal disorders | Sputum increased | 5 | 11.86(4.93,28.51) | 11.85(49.64) | 3.57(0.90) |
| Cardiac disorders | Pulseless electrical activity | 4 | 11.65(4.37,31.07) | 11.65(38.91) | 3.54(0.60) |
| Hepatobiliary disorders | Venoocclusive liver disease | 5 | 11.50(4.78,27.65) | 11.50(47.88) | 3.52(0.88) |
| General disorders and administration site conditions | Systemic inflammatory response syndrome | 3 | 11.02(3.55,34.18) | 11.01(27.29) | 3.46(0.21) |
| Investigations | Blood pressure systolic decreased | 4 | 10.51(3.94,28.03) | 10.51(34.40) | 3.39(0.56) |
| Metabolism and nutrition disorders | Hypoalbuminaemia | 5 | 10.06(4.18,24.17) | 10.05(40.72) | 3.33(0.82) |
| Hepatobiliary disorders | Hepatotoxicity | 17 | 9.65(5.99,15.53) | 9.63(131.40) | 3.27(2.02) |
| Blood and lymphatic system disorders | Autoimmune haemolytic anaemia | 3 | 9.50(3.06,29.48) | 9.50(22.80) | 3.25(0.16) |
| Hepatobiliary disorders | Jaundice cholestatic | 4 | 9.49(3.56,25.31) | 9.49(30.36) | 3.25(0.52) |
| Hepatobiliary disorders | Hepatic function abnormal | 96 | 9.41(7.69,11.51) | 9.30(711.70) | 3.22(2.80) |
| Renal and urinary disorders | Nephropathy toxic | 9 | 8.80(4.58,16.93) | 8.79(62.15) | 3.14(1.39) |
| Investigations | Gamma-glutamyltransferase increased | 24 | 8.62(5.77,12.86) | 8.59(160.95) | 3.10(2.14) |
| Skin and subcutaneous tissue disorders | Toxic epidermal necrolysis | 19 | 8.60(5.48,13.50) | 8.58(127.27) | 3.10(1.99) |
| Respiratory, thoracic and mediastinal disorders | Respiratory failure | 43 | 8.40(6.22,11.33) | 8.35(278.41) | 3.06(2.40) |
| Respiratory, thoracic and mediastinal disorders | Pulmonary alveolar haemorrhage | 3 | 8.36(2.69,25.92) | 8.35(19.41) | 3.06(0.11) |
| Investigations | Transaminases increased | 29 | 8.30(5.76,11.95) | 8.27(185.35) | 3.05(2.21) |
| Skin and subcutaneous tissue disorders | Drug eruption | 27 | 8.16(5.59,11.91) | 8.13(168.94) | 3.02(2.15) |
| Hepatobiliary disorders | Liver injury | 20 | 8.06(5.20,12.50) | 8.04(123.30) | 3.01(1.96) |
| Respiratory, thoracic and mediastinal disorders | Acute respiratory distress syndrome | 11 | 7.95(4.40,14.37) | 7.94(66.75) | 2.99(1.50) |
| Vascular disorders | Haemodynamic instability | 3 | 7.71(2.48,23.90) | 7.70(17.49) | 2.94(0.08) |
| Gastrointestinal disorders | Large intestine perforation | 4 | 7.37(2.76,19.64) | 7.36(21.99) | 2.88(0.40) |
| Gastrointestinal disorders | Ileus paralytic | 3 | 7.34(2.37,22.78) | 7.34(16.43) | 2.88(0.06) |
| Investigations | Hepatic enzyme increased | 65 | 7.31(5.73,9.34) | 7.26(350.98) | 2.86(2.37) |
| Cardiac disorders | Torsade de pointes | 5 | 7.22(3.00,17.35) | 7.22(26.76) | 2.85(0.65) |
| Nervous system disorders | Posterior reversible encephalopathy syndrome | 5 | 7.18(2.99,17.27) | 7.18(26.59) | 2.84(0.64) |
| Respiratory, thoracic and mediastinal disorders | Pneumothorax | 8 | 6.93(3.46,13.86) | 6.92(40.52) | 2.79(1.10) |
| Metabolism and nutrition disorders | Failure to thrive | 3 | 6.80(2.19,21.09) | 6.80(14.83) | 2.76(0.03) |
| Hepatobiliary disorders | Acute hepatic failure | 6 | 6.76(3.03,15.05) | 6.75(29.40) | 2.76(0.80) |
| Skin and subcutaneous tissue disorders | Macule | 3 | 6.52(2.10,20.21) | 6.51(13.99) | 2.70(0.01) |
| Metabolism and nutrition disorders | Hypophosphataemia | 4 | 6.45(2.42,17.20) | 6.45(18.41) | 2.69(0.33) |
| Renal and urinary disorders | Renal impairment | 52 | 6.37(4.85,8.37) | 6.33(233.75) | 2.66(2.13) |
| Hepatobiliary disorders | Hepatic failure | 15 | 6.30(3.80,10.46) | 6.29(66.74) | 2.65(1.52) |
| Hepatobiliary disorders | Hepatic cytolysis | 13 | 6.28(3.65,10.83) | 6.27(57.63) | 2.65(1.42) |
| Gastrointestinal disorders | Gastrointestinal inflammation | 3 | 6.23(2.01,19.33) | 6.23(13.16) | 2.64(-0.01) |
| Blood and lymphatic system disorders | Febrile neutropenia | 45 | 5.97(4.45,8.00) | 5.94(185.04) | 2.57(2.00) |
| Metabolism and nutrition disorders | Hypokalaemia | 39 | 5.57(4.07,7.64) | 5.55(145.58) | 2.47(1.86) |
| Investigations | Blood alkaline phosphatase increased | 16 | 5.57(3.41,9.09) | 5.56(59.81) | 2.47(1.43) |
| Gastrointestinal disorders | Oral pain | 11 | 5.34(2.96,9.65) | 5.34(38.77) | 2.42(1.14) |
| Investigations | Blood urea increased | 10 | 5.23(2.81,9.72) | 5.22(34.13) | 2.38(1.04) |
| Hepatobiliary disorders | Cholestasis | 8 | 5.15(2.58,10.31) | 5.15(26.73) | 2.36(0.85) |
| Respiratory, thoracic and mediastinal disorders | Pleural effusion | 23 | 5.12(3.40,7.71) | 5.11(76.04) | 2.35(1.53) |
| Investigations | Platelet count decreased | 46 | 4.92(3.68,6.58) | 4.90(142.92) | 2.29(1.76) |
| Investigations | Liver function test increased | 10 | 4.87(2.62,9.06) | 4.87(30.73) | 2.28(0.98) |
| Skin and subcutaneous tissue disorders | Toxic skin eruption | 6 | 4.86(2.18,10.81) | 4.85(18.35) | 2.28(0.55) |
| Respiratory, thoracic and mediastinal disorders | Acute respiratory failure | 8 | 4.77(2.38,9.53) | 4.76(23.77) | 2.25(0.78) |
| Blood and lymphatic system disorders | Pancytopenia | 29 | 4.75(3.30,6.84) | 4.74(85.58) | 2.24(1.55) |
| Skin and subcutaneous tissue disorders | Rash morbilliform | 6 | 4.56(2.05,10.16) | 4.56(16.66) | 2.19(0.50) |
| Blood and lymphatic system disorders | Thrombocytopenia | 97 | 4.50(3.68,5.50) | 4.45(260.50) | 2.15(1.81) |
| Psychiatric disorders | Eating disorder | 7 | 4.38(2.09,9.19) | 4.38(18.24) | 2.13(0.60) |
| Blood and lymphatic system disorders | Eosinophilia | 12 | 4.36(2.48,7.69) | 4.36(31.07) | 2.12(0.99) |
| Vascular disorders | Shock | 9 | 4.23(2.20,8.13) | 4.23(22.17) | 2.08(0.76) |
| Nervous system disorders | Altered state of consciousness | 8 | 4.22(2.11,8.45) | 4.22(19.66) | 2.08(0.67) |
| Investigations | Blood creatinine increased | 26 | 4.01(2.73,5.90) | 4.00(58.62) | 2.00(1.29) |
| Cardiac disorders | Cardiac arrest | 25 | 3.17(2.14,4.70) | 3.17(37.07) | 1.66(0.98) |
| Renal and urinary disorders | Acute kidney injury | 59 | 3.06(2.36,3.95) | 3.04(80.93) | 1.60(1.18) |

SOC, system organ class; PT, preferred term; ROR, reporting odds ratio; CI, confidence interval; PRR, proportional reporting ratio; χ2, chi-squared; IC, information component; IC025, the lower limit of 95%CI of the IC.

Table S11 Signal strength of adverse events associated with anidulafungin at the PT level ranked by ROR in Vigiaccess.

| SOC | PT | N | ROR (95% CI) | PRR (χ^2^) | IC (IC025) |
| --- | --- | --- | --- | --- | --- |
| Investigations | Cardiac index decreased | 3 | 1621.61(514.02,5115.77) | 1619.34(4713.39) | 10.62(0.53) |
| Hepatobiliary disorders | Hepatotoxicity | 55 | 112.62(86.14,147.23) | 109.75(5916.45) | 6.78(4.83) |
| Vascular disorders | Haemodynamic instability | 6 | 54.38(24.39,121.22) | 54.23(313.17) | 5.76(1.56) |
| Hepatobiliary disorders | Acute hepatic failure | 11 | 43.80(24.21,79.22) | 43.58(457.29) | 5.44(2.42) |
| Metabolism and nutrition disorders | Hypomagnesaemia | 10 | 33.84(18.18,63.00) | 33.69(317.03) | 5.07(2.21) |
| General disorders and administration site conditions | Multiple organ dysfunction syndrome | 23 | 30.75(20.38,46.38) | 30.43(654.47) | 4.93(3.18) |
| Respiratory, thoracic and mediastinal disorders | Acute pulmonary oedema | 3 | 30.49(9.82,94.64) | 30.45(85.39) | 4.93(0.42) |
| Metabolism and nutrition disorders | Hypokalaemia | 47 | 24.04(18.01,32.10) | 23.54(1014.72) | 4.56(3.58) |
| Respiratory, thoracic and mediastinal disorders | Choking sensation | 3 | 19.03(6.13,59.06) | 19.01(51.16) | 4.25(0.34) |
| Investigations | Liver function test abnormal | 18 | 18.87(11.86,30.01) | 18.72(301.89) | 4.23(2.61) |
| Hepatobiliary disorders | Hepatic failure | 12 | 17.80(10.09,31.39) | 17.70(189.13) | 4.15(2.15) |
| Investigations | Liver function test increased | 10 | 17.20(9.24,32.02) | 17.13(151.84) | 4.10(1.92) |
| Metabolism and nutrition disorders | Hypophosphataemia | 3 | 17.04(5.49,52.88) | 17.01(45.21) | 4.09(0.32) |
| Investigations | Blood bilirubin increased | 13 | 16.31(9.46,28.14) | 16.22(185.68) | 4.02(2.18) |
| Hepatobiliary disorders | Cholestasis | 7 | 15.90(7.57,33.39) | 15.85(97.36) | 3.99(1.45) |
| Investigations | Platelet count increased | 5 | 15.86(6.60,38.16) | 15.83(69.46) | 3.98(1.01) |
| Investigations | Hepatic enzyme increased | 32 | 12.74(8.99,18.07) | 12.57(341.07) | 3.65(2.71) |
| Respiratory, thoracic and mediastinal disorders | Respiratory failure | 18 | 12.40(7.79,19.71) | 12.30(186.95) | 3.62(2.28) |
| Nervous system disorders | Status epilepticus | 3 | 12.22(3.94,37.92) | 12.20(30.85) | 3.61(0.24) |
| Investigations | Transaminases increased | 12 | 12.10(6.86,21.34) | 12.04(121.48) | 3.59(1.90) |
| Hepatobiliary disorders | Hypertransaminasaemia | 3 | 11.18(3.60,34.70) | 11.17(27.76) | 3.48(0.21) |
| Investigations | Blood alkaline phosphatase increased | 9 | 11.04(5.74,21.25) | 11.00(81.82) | 3.46(1.54) |
| Cardiac disorders | Ventricular fibrillation | 3 | 9.78(3.15,30.36) | 9.77(23.62) | 3.29(0.17) |
| General disorders and administration site conditions | Hypothermia | 3 | 9.74(3.14,30.23) | 9.73(23.49) | 3.28(0.17) |
| Blood and lymphatic system disorders | Eosinophilia | 7 | 8.97(4.27,18.84) | 8.95(49.42) | 3.16(1.14) |
| Hepatobiliary disorders | Liver injury | 6 | 8.51(3.82,18.96) | 8.49(39.63) | 3.08(0.94) |
| Respiratory, thoracic and mediastinal disorders | Tachypnoea | 7 | 8.38(3.99,17.60) | 8.35(45.33) | 3.06(1.10) |
| Blood and lymphatic system disorders | Coagulopathy | 5 | 8.33(3.46,20.03) | 8.31(32.16) | 3.05(0.73) |
| Hepatobiliary disorders | Hyperbilirubinaemia | 4 | 7.76(2.91,20.70) | 7.75(23.52) | 2.95(0.43) |
| Respiratory, thoracic and mediastinal disorders | Acute respiratory distress syndrome | 3 | 7.63(2.46,23.68) | 7.62(17.26) | 2.93(0.08) |
| Investigations | Blood potassium decreased | 5 | 7.59(3.16,18.26) | 7.58(28.55) | 2.92(0.67) |
| Investigations | Blood urea increased | 4 | 7.36(2.76,19.63) | 7.35(21.94) | 2.88(0.40) |
| Hepatobiliary disorders | Hepatocellular injury | 3 | 7.01(2.26,21.75) | 7.00(15.43) | 2.81(0.04) |
| Respiratory, thoracic and mediastinal disorders | Bronchospasm | 8 | 6.95(3.47,13.92) | 6.93(40.61) | 2.79(1.10) |
| Vascular disorders | Circulatory collapse | 5 | 6.45(2.68,15.51) | 6.43(22.95) | 2.69(0.58) |
| Respiratory, thoracic and mediastinal disorders | Laryngeal oedema | 3 | 6.40(2.06,19.86) | 6.39(13.64) | 2.68(0.00) |
| Skin and subcutaneous tissue disorders | Toxic epidermal necrolysis | 4 | 6.37(2.39,16.98) | 6.36(18.05) | 2.67(0.33) |
| Metabolism and nutrition disorders | Hyperkalaemia | 7 | 6.19(2.95,13.00) | 6.17(30.37) | 2.63(0.88) |
| Respiratory, thoracic and mediastinal disorders | Hypoxia | 6 | 5.90(2.65,13.14) | 5.88(24.33) | 2.56(0.70) |
| General disorders and administration site conditions | Generalised oedema | 4 | 5.88(2.20,15.68) | 5.87(16.17) | 2.55(0.28) |
| Investigations | Aspartate aminotransferase increased | 11 | 5.65(3.12,10.22) | 5.63(41.87) | 2.49(1.19) |
| Blood and lymphatic system disorders | Thrombocytopenia | 34 | 5.56(3.97,7.81) | 5.49(125.26) | 2.46(1.79) |
| Immune system disorders | Anaphylactic reaction | 22 | 5.55(3.65,8.45) | 5.50(81.21) | 2.46(1.60) |
| Vascular disorders | Cyanosis | 8 | 5.46(2.73,10.93) | 5.44(29.03) | 2.44(0.90) |
| Blood and lymphatic system disorders | Pancytopenia | 9 | 5.19(2.70,9.99) | 5.17(30.32) | 2.37(0.95) |
| Investigations | C-reactive protein increased | 4 | 5.11(1.91,13.62) | 5.10(13.19) | 2.35(0.19) |
| Skin and subcutaneous tissue disorders | Drug reaction with eosinophilia and systemic symptoms | 4 | 5.08(1.90,13.55) | 5.07(13.08) | 2.34(0.19) |
| Investigations | Alanine aminotransferase increased | 12 | 5.06(2.87,8.93) | 5.04(38.91) | 2.33(1.14) |
| Investigations | Gamma-glutamyltransferase increased | 4 | 5.04(1.89,13.45) | 5.04(12.94) | 2.33(0.19) |
| Investigations | Blood creatinine increased | 9 | 4.89(2.54,9.41) | 4.87(27.74) | 2.29(0.90) |
| Investigations | Oxygen saturation decreased | 7 | 4.84(2.31,10.17) | 4.83(21.26) | 2.27(0.68) |
| Musculoskeletal and connective tissue disorders | Rhabdomyolysis | 5 | 4.54(1.89,10.91) | 4.53(13.75) | 2.18(0.33) |
| Hepatobiliary disorders | Jaundice | 5 | 3.99(1.66,9.59) | 3.98(11.17) | 1.99(0.23) |
| Respiratory, thoracic and mediastinal disorders | Pulmonary oedema | 4 | 3.85(1.44,10.27) | 3.84(8.42) | 1.94(0.00) |
| Immune system disorders | Anaphylactic shock | 11 | 3.77(2.08,6.81) | 3.75(22.22) | 1.91(0.77) |
| Hepatobiliary disorders | Hepatic function abnormal | 11 | 3.76(2.08,6.80) | 3.75(22.19) | 1.91(0.77) |
| Vascular disorders | Hypotension | 27 | 3.53(2.42,5.16) | 3.50(48.35) | 1.81(1.13) |
| Skin and subcutaneous tissue disorders | Erythema | 39 | 3.22(2.34,4.42) | 3.18(58.53) | 1.67(1.13) |
| Cardiac disorders | Tachycardia | 18 | 3.17(2.00,5.05) | 3.16(26.57) | 1.66(0.84) |
| Vascular disorders | Flushing | 17 | 3.10(1.92,4.99) | 3.08(23.97) | 1.62(0.78) |
| Renal and urinary disorders | Renal failure | 11 | 3.02(1.67,5.46) | 3.01(14.78) | 1.59(0.53) |
| Cardiac disorders | Bradycardia | 6 | 2.69(1.21,5.99) | 2.68(6.34) | 1.42(0.02) |
| Cardiac disorders | Cardiac arrest | 6 | 2.68(1.20,5.97) | 2.67(6.29) | 1.42(0.02) |
| Nervous system disorders | Seizure | 14 | 2.35(1.39,3.97) | 2.34(10.73) | 1.22(0.35) |
| Renal and urinary disorders | Acute kidney injury | 12 | 2.18(1.24,3.85) | 2.17(7.63) | 1.12(0.19) |
| Respiratory, thoracic and mediastinal disorders | Dyspnoea | 50 | 2.09(1.58,2.77) | 2.07(27.81) | 1.05(0.61) |
| Musculoskeletal and connective tissue disorders | Back pain | 15 | 2.09(1.26,3.47) | 2.08(8.48) | 1.06(0.24) |

SOC, system organ class; PT, preferred term; ROR, reporting odds ratio; CI, confidence interval; PRR, proportional reporting ratio; χ2, chi-squared; IC, information component; IC025, the lower limit of 95%CI of the IC.
